# Improving Assessment of Drug Safety Through Proteomics: Early Detection and Mechanistic Characterization of the Unforeseen Harmful Effects of Torcetrapib

**DOI:** 10.1161/CIRCULATIONAHA.117.028213

**Published:** 2018-03-05

**Authors:** Stephen A. Williams, Ashwin C. Murthy, Robert K. DeLisle, Craig Hyde, Anders Malarstig, Rachel Ostroff, Sophie J. Weiss, Mark R. Segal, Peter Ganz

**Affiliations:** 1 1 SomaLogic, Inc., Boulder, CO (S.A.W., R.K.D., R.O., S.J.W.).; 2 2 Department of Medicine (A.C.M., P.G.); 3 3 Department of Epidemiology and Biostatistics (M.R.S.); 4 4 University of California, San Francisco. Pfizer Inc., Worldwide Research and Development, Groton, CT (C.H.).; 5 5 Pfizer Inc., Worldwide Research and Development, Stockholm, Sweden (A.M.).; 6 6 Division of Cardiology, Zuckerberg San Francisco General Hospital, CA (P.G.).

**Keywords:** aptamers, biomarkers, peptides, precision medicine, prescription drugs, proteins, proteomics

## Abstract

**Background:**

Early detection of adverse effects of novel therapies and understanding of their mechanisms could improve the safety and efficiency of drug development. We have retrospectively applied large-scale proteomics to blood samples from ILLUMINATE (Investigation of Lipid Level Management to Understand its Impact in Atherosclerotic Events), a trial of torcetrapib (a cholesterol ester transfer protein inhibitor), that involved 15 067 participants at high cardiovascular risk. ILLUMINATE was terminated at a median of 550 days because of significant absolute increases of 1.2% in cardiovascular events and 0.4% in mortality with torcetrapib. The aims of our analysis were to determine whether a proteomic analysis might reveal biological mechanisms responsible for these harmful effects and whether harmful effects of torcetrapib could have been detected early in the ILLUMINATE trial with proteomics.

**Methods:**

A nested case-control analysis of paired plasma samples at baseline and at 3 months was performed in 249 participants assigned to torcetrapib plus atorvastatin and 223 participants assigned to atorvastatin only. Within each treatment arm, cases with events were matched to controls 1:1. Main outcomes were a survey of 1129 proteins for discovery of biological pathways altered by torcetrapib and a 9-protein risk score validated to predict myocardial infarction, stroke, heart failure, or death.

**Results:**

Plasma concentrations of 200 proteins changed significantly with torcetrapib. Their pathway analysis revealed unexpected and widespread changes in immune and inflammatory functions, as well as changes in endocrine systems, including in aldosterone function and glycemic control. At baseline, 9-protein risk scores were similar in the 2 treatment arms and higher in participants with subsequent events. At 3 months, the absolute 9-protein derived risk increased in the torcetrapib plus atorvastatin arm compared with the atorvastatin-only arm by 1.08% (*P*=0.0004). Thirty-seven proteins changed in the direction of increased risk of 49 proteins previously associated with cardiovascular and mortality risk.

**Conclusions:**

Heretofore unknown effects of torcetrapib were revealed in immune and inflammatory functions. A protein-based risk score predicted harm from torcetrapib within just 3 months. A protein-based risk assessment embedded within a large proteomic survey may prove to be useful in the evaluation of therapies to prevent harm to patients.

**Clinical Trial Registration:**

URL: https://www.clinicaltrials.gov. Unique identifier: NCT00134264.

## Abstract 2

Supplemental Digital Content is available in the text.

**Editorial, see p 1011**

**Clinical Perspective**

**What Is New?**

- Early detection of harmful effects of novel drug treatments and their mechanistic understanding could improve the safety of drug development.
- ILLUMINATE (Investigation of Lipid Level Management to Understand its Impact in Atherosclerotic Events) was a trial of torcetrapib, a cholesterol ester transfer protein inhibitor, which raised high-density lipoprotein cholesterol and lowered low-density lipoprotein cholesterol. The trial was terminated because of increases in cardiovascular events and mortality.
- In a retrospective analysis of baseline and on-treatment blood samples from ILLUMINATE, a large-scale proteomic analysis detected harm from torcetrapib at 3 months of treatment, before it became apparent clinically.
- Proteomic analysis revealed unexpected alterations in inflammation and immunity by torcetrapib and explained the previously reported activation of aldosterone.

**What Are the Clinical Implications?**

- A longitudinal survey of the proteome in blood samples can provide an early warning of unexpected harm from drug therapies and inform responsible mechanisms.
- Proteomics, a tool of precision medicine, may prove to be useful in improving the safety and efficiency of drug development.

In 2006, the results of the 15 067–patient ILLUMINATE trial (Investigation of Lipid Level Management to Understand its Impact in Atherosclerotic Events)^1^ were described as a “nightmare created by unintended pharmacological effects” of torcetrapib.^2^ Torcetrapib, a cholesteryl ester transfer protein inhibitor, given alongside atorvastatin, raised high-density lipoprotein (HDL) cholesterol by a remarkable 72% and reduced low-density lipoprotein (LDL) cholesterol by 25%. Despite these favorable changes in lipid profile, the trial was terminated after a median follow-up of 550 days because of significant absolute increases of 1.2% in cardiovascular events and 0.4% in all-cause deaths in the torcetrapib plus atorvastatin arm compared with the atorvastatin-only arm.^1^ Aside from the harm to patients and the reported $1 billion cost of the failed torcetrapib development program,^3^ the misleading results from the previously trusted cardiovascular risk biomarkers LDL and HDL cholesterol have led to a view that these (and possibly other) biomarkers are not reliable surrogates for meaningful clinical outcomes.^4,5^ The consequences of this view are clinical drug development programs that are potentially harmful to patients, slow to complete, and expensive. Indeed, the absence of reliable biomarkers to predict adverse drug effects in a timely manner and before harm to study participants accrues is currently regarded by the US Food and Drug Administration as a key obstacle to the pace of innovation in drug development across all diseases.^6^

Proteins are key regulators of biological processes and relate to the risk of diseases and their clinical outcomes.^7,8^ The field of proteomics has matured over the past 20 years, and it is now poised to have a transformative impact on cardiovascular health and disease.^9^ Technologies have been developed that can readily measure the levels of hundreds or even thousands of proteins in a small sample of blood.^8,9^ The objectives of this study were to use one such technology, modified aptamers,^8,10–13^ to measure plasma proteins in a nested case-control subset of patients from ILLUMINATE to determine whether mechanistic insights into the harmful effects of torcetrapib could be discerned from an analysis of 1129 plasma proteins with the tools of pathway analysis and to determine whether a previously validated 9-protein cardiovascular risk score^8^ could have detected changes in cardiovascular and all-cause mortality risk over the first 3 months of assigned treatment with torcetrapib plus atorvastatin compared with atorvastatin only.

## Methods

### Study Population

ILLUMINATE was a prospective, multicenter, placebo-controlled trial that randomized a total of 15 067 patients between August 23, 2004, and December 28, 2005.^1^ Men and women between the ages of 45 and 75 years were eligible to participate if they had a history of cardiovascular disease (myocardial infarction, stroke, acute coronary syndrome, unstable angina, peripheral vascular disease, and cardiac revascularization) or type 2 diabetes mellitus without overt cardiovascular disease. During a run-in period of 4 to 10 weeks, patients underwent lifestyle counseling and uptitration of atorvastatin, as needed, at 2-week intervals to achieve an LDL cholesterol level <100 mg/dL. The trial was terminated prematurely on December 2, 2006, because of significant absolute increases of 1.2% in cardiovascular events and 0.4% in mortality in patients receiving torcetrapib, with a median follow-up of 550 days and the longest follow-up of 880 days.^1^ An institutional review board at each ILLUMINATE center approved the study protocol, and patients provided written informed consent.^1^

### Plasma Samples and Study Design

**Figure 1.**
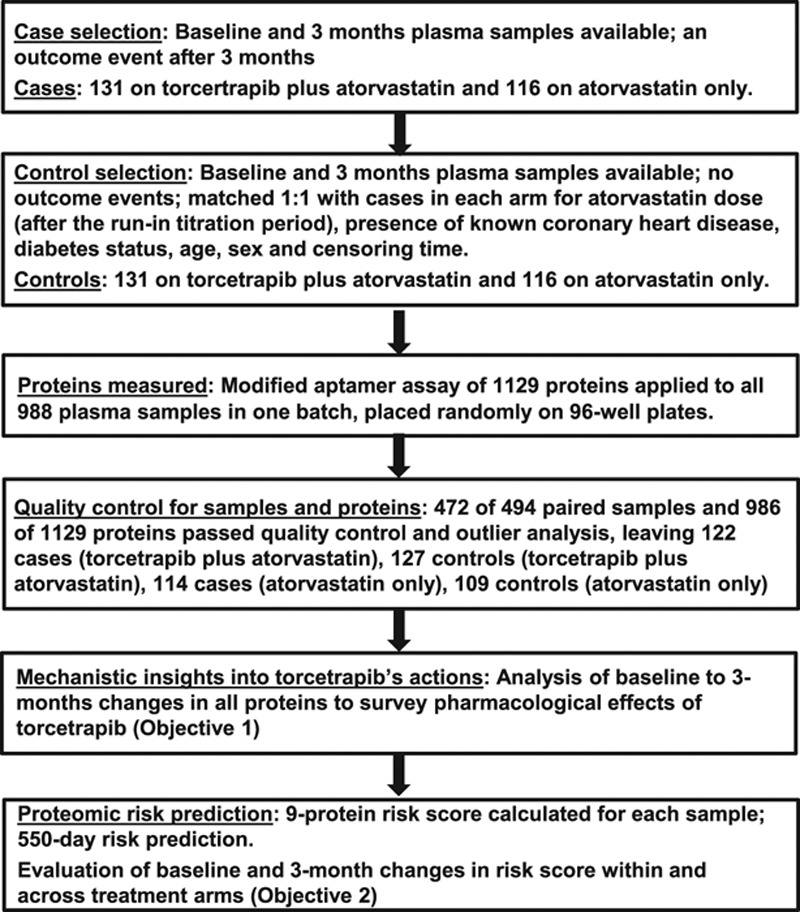
**Flowchart of samples, study participants, and analytic processes for the evaluation of torcetrapib.**

Plasma (EDTA) samples from the study participants were biobanked by the ILLUMINATE study sponsor (Pfizer Inc.). We used a nested case-control design^14^ to maximize the power to detect an effect by including all the participants with outcome events and available plasma samples. Paired plasma samples obtained at baseline and at 3 months from a total of 494 study participants were used to determine within-participant changes in plasma proteins during the first 3 months of randomized treatment. All individuals selected as cases had a first event after the 3-month sample. Cases in this analysis were selected with the same definition of events used previously to derive and validate the 9-protein risk model (myocardial infarction, stroke/transient ischemic attack, hospitalization for heart failure, or all-cause death).^8^ Although not identical to the primary outcome of ILLUMINATE, each of the end points reported in the present analysis was adjudicated by a committee of ILLUMINATE as a primary or a secondary end-point of the trial.^1^ For each case, a matched control participant was selected within each treatment arm on the basis of the following baseline characteristics: atorvastatin dose (after the run-in uptitration period), presence of known coronary heart disease, diabetes status, age, sex, and censoring time. The baseline plasma samples were obtained before the participant was assigned treatment, after the run-in atorvastatin uptitration. The study flowchart is shown in Figure 1.

### Quantification of Proteins in Human Plasma

In total, 988 plasma samples were analyzed by a modified aptamer assay.^8^ In brief, each of the 1129 individual proteins measured has its binding reagent made of chemically modified DNA, referred to as a modified aptamer. The assay version was nearly identical to that described previously to derive a 9-protein cardiovascular risk score.^8^ Each sample of plasma was incubated with the mixture of modified aptamers to generate modified aptamer-protein complexes under equilibrium conditions. Unbound modified aptamers and unbound or nonspecifically bound proteins were eliminated by 2 bead-based immobilization steps. After elution of the modified aptamers from the target protein, the fluorescently labeled modified aptamers were directly quantified on a hybridization array (Agilent Technologies). Calibrators were included so that the degree of fluorescence was a consistent reflection of protein concentration. The median intra-assay, interassay, and total coefficients of variation for the 1129 proteins are all <4%. The distribution of coefficients of variation across the 1129 proteins measured and coefficients of variation for each of the proteins within the 9-protein prognostic model^8^ are provided in Table I in the online-only Data Supplement. All of the 1129 protein targets are above the limit of detection as defined by the Clinical and Laboratory Standards Institute.^15^ Of the 1129 proteins measured, 143 did not pass the interrun calibration quality control metrics in at least 1 of the thirteen 96-well plates that were run and were thus excluded from further analysis (detailed information on exclusion of proteins is provided in Method I in the online-only Data Supplement). One sample of the 988 samples was not analyzed because of an error. Twenty-two samples did not pass the intrarun normalization quality control metrics and were excluded from analysis. In 2 samples, 10% of the protein measurements exceeded an outlier threshold defined as the median protein signal level±6 median absolute deviations and 5 times higher or lower than the median protein signal level. A further 19 samples were removed from the analysis because their pair was not run, did not pass the quality control threshold, or exceeded the aforementioned outlier threshold. Overall, the exclusions from the protein assay and sample quality control metrics resulted in an assessment of 986 of the 1129 assayed proteins, 249 pairs of samples in the torcetrapib plus atorvastatin arm, and 223 pairs in the atorvastatin-only arm. The flow diagram is shown in Figure 1.

### Statistical Methods

All samples were placed randomly on 96-well plates, run in a single batch, and normalized against protein calibrator samples included on each plate.^8,11^ Assay personnel were unaware of the treatment or the clinical outcome. Because the assay version was slightly more recent compared with that used to derive and validate the 9-protein model,^8^ 60 bridging samples from the earlier analyses were used as protein standards to align the fluorescence readouts of the same proteins in the same samples across the different assay versions (Method II in the online-only Data Supplement).

For study objective 1, to discern the biological mechanisms of torcetrapib by assessing treatment-associated changes in plasma protein concentrations and organizing differentially expressed proteins according to biological pathways, differences between the baseline and 3-month samples were expressed as log2 ratios for each of the 986 proteins measured that passed quality control. Significance values were calculated within each treatment arm with the Wilcoxon signed-rank test for paired comparisons corrected for a false discovery rate in 986 measurements and across treatment arms with the Wilcoxon signed-rank test for unpaired comparisons. Proteins with a false discovery rate–adjusted value of *P*<0.05 were considered statistically significant for inclusion in the pathway analysis.

Ingenuity Pathway Analysis (IPA) was used to cluster differentially expressed proteins at 3 months compared with baseline into pathways and functional groups in the torcetrapib plus atorvastatin treatment arm (IPA content version 28820210, release date September 24, 2016, Ingenuity Systems Inc, Redwood City, CA; www.ingenuity.com). A pathway analysis was not performed within the atorvastatin-only treatment arm because only a few proteins changed over 3 months. For those modified aptamers that had multiple Uniprot identifications associated with 1 result, only the first listed Uniprot identification was used in the pathway analysis. The Fisher right-tailed exact test was used to calculate a *P* value to determine the probability that the association of the differently expressed proteins in the measured data set, and the pathway is explained by chance alone. Of the 986 proteins measured, 980 were recognized by IPA and used as the background reference. The pathway analysis results obtained by IPA, a commercial system, were confirmed by 2 open-source pathway analysis systems, Reactome Pathway Database (http://reactome.org/) and Database for Annotation, Visualization and Integrated Discovery (DAVID) Bioinformatics resource (https://david.ncifcrf.gov/).

For study objective 2, to determine whether a previously validated 9-protein cardiovascular risk score could detect changes in risk over the first 3 months of assigned treatment with torcetrapib plus atorvastatin compared with atorvastatin only, the 9-protein risk score^8^ was calculated without recalibration to this study population. This 9-protein risk score was previously constructed by a rigorous bioinformatics process, starting from a total of 1130 plasma proteins.^8^ The model consists of the following 9 proteins (in rank order of their contribution to risk calculation): ANGPT2 (angiopoietin-2), GDF11/8 (growth differentiation factor 11/8), C7 (complement 7), SERPINF2 (α2-antiplasmin), CCL18 (chemokine [C-C motif] ligand 18), ANGPTL4 (angiopoietin-related protein 4), SERPINA3 (α1-antichymotrypsin complex), MMP12 (matrix metalloproteinase-12), and TNNI3 (troponin I).^8^ The prognostic risk score is as follows:


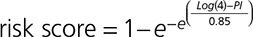


where the prognostic index (PI) combines the measurements of the 9 proteins as follows:


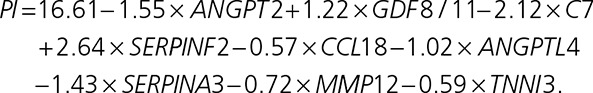


The published risk score allows the calculation of probabilities of events for any specified time horizon.^8^ For this application, an output of 550 days, the median duration of treatment for patients in ILLUMINATE,^1^ was selected so that the change in observed event rate in ILLUMINATE could be compared with the prediction of the 9-protein risk model.

For study objective 2, the analysis combined all participants with or without events within each of the 2 treatment arms. Changes in the 9-protein risk score between baseline and 3 months were compared within each treatment arm with the paired Wilcoxon signed-rank test for paired comparisons. Changes in risk score in cases compared with controls within each treatment arm were also evaluated with the unpaired Wilcoxon rank-sum test. *P* values were not corrected for multiple comparisons. The Ansari-Bradley test^16^ was applied to discern whether torcetrapib-associated changes in the 9-protein risk score represented a single distribution and thus whether torcetrapib affected all individuals exposed.

Although most participants in the ILLUMINATE trial had known coronary heart disease and therefore matched the population on which the 9-protein model was derived, the study protocol also allowed enrollment of diabetics without known coronary heart disease, who represented 19% of the ILLUMINATE study population.^1^ Because no differences in distributions of protein-based risk scores could be discerned between participants with coronary heart disease and the larger group of participants that also included diabetics without coronary heart disease (Figure I in the online-only Data Supplement), it was the larger group that was used in the analyses.

For comparative purposes, to clarify how reported torcetrapib-associated increases in blood pressure in ILLUMINATE^1^ balance out against improvements in HDL and LDL cholesterol levels for cardiovascular risk prediction, the Framingham secondary event risk prediction score was also calculated for the identical time horizon.^17^ This Framingham risk score is applicable to patients with established cardiovascular disease such as those enrolled in the ILLUMINATE trial.^17^

All pathway analyses were performed at the University of California, San Francisco (by A.C.M. and P.G.). All other statistical analyses were performed initially at SomaLogic, Inc., (by S.J.W.) and replicated at University of California, San Francisco (by M.R.S.).

All statistical computing was performed with the R Language for Statistical Computing, version 3.3.1 at SomaLogic, Inc., and version 3.2.1 at the University of California, San Francisco.

## Results

### Study Population Characteristics

The baseline characteristics of the study population after the atorvastatin uptitration run-in period are shown in Table 1. The 2 treatment arms were well matched. Within each arm, patients with outcome events (cases) were more likely to have lower estimated glomerular filtration rates and higher diastolic blood pressure than patients without events (controls). Compared with the entire ILLUMINATE study population,^1^ the participants in this analysis were older and more likely to be male and diabetic (data not shown). These differences are expected because they reflect the nested case-control study design with half of the participants experiencing outcomes events (cases) to which the control participants were matched for high-risk cardiovascular characteristics, particularly age, sex, and diabetes status.

**Table 1.**
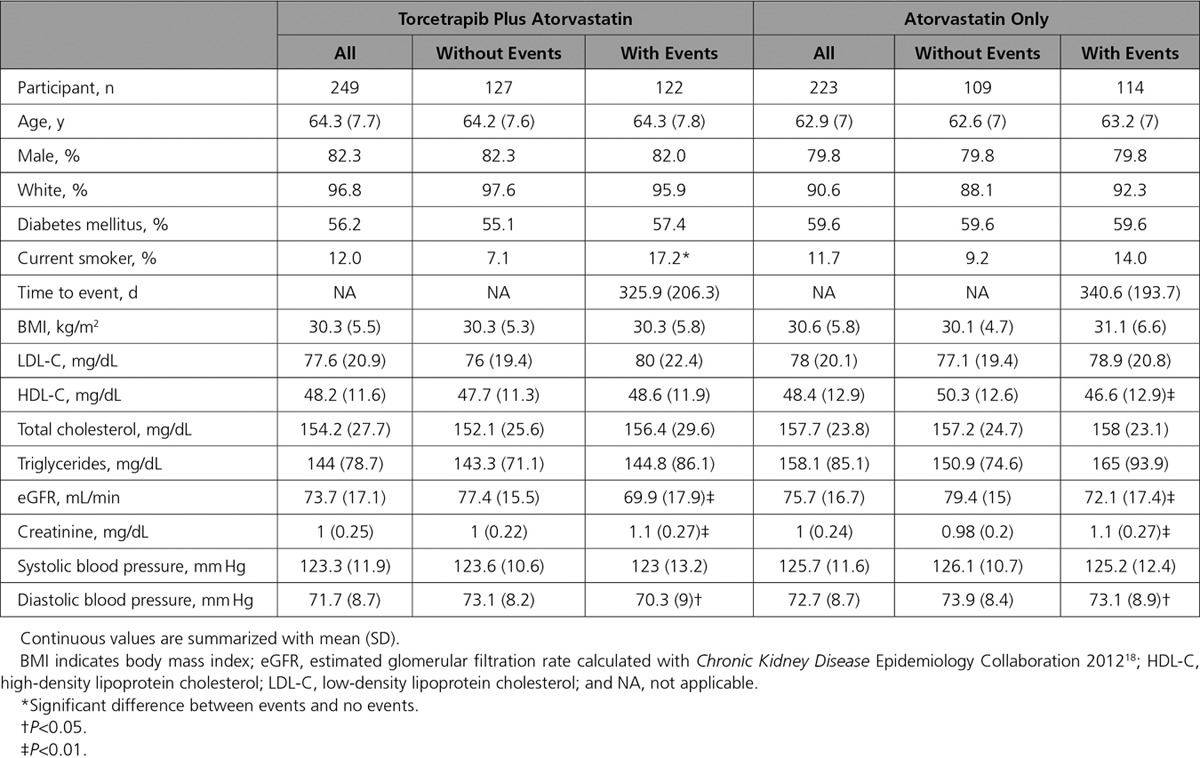
Baseline Characteristics of the Case-Control Study Population

### Mechanistic Insights Into the Effects on Torcetrapib Based on a Proteomic Survey and Pathway Analysis

**Figure 2.**
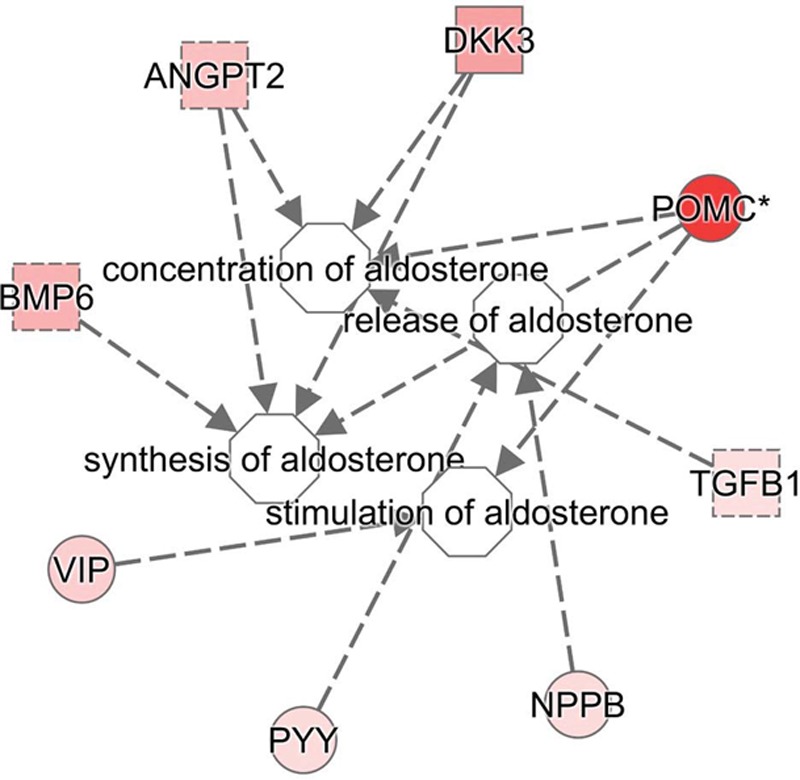
**Proteins significantly altered by torcetrapib associated with aldosterone synthesis or function.** Nodes represent gene symbol name, corresponding to protein measured. Degree of intensity of the node color (red) indicates degree of significance of the false discovery rate *P* value. Dashed lines correspond to the implicated function of the given proteins according to the Ingenuity Pathway Analysis knowledge bank. Node shapes denote cytokine (□), growth factor (□), and other (○). ANGPT2 indicates angiopoietin-2; BMP6, bone morphometric protein 6; DKK3, Dickkopf-related protein 3; NPPB, natriuretic peptide B; POMC, pro-opiomelanocortin; PYY, peptide YY; and TGFB1, transforming growth factor-β1. *Given protein was represented in the modified aptamer assay more than once.

**Figure 3.**
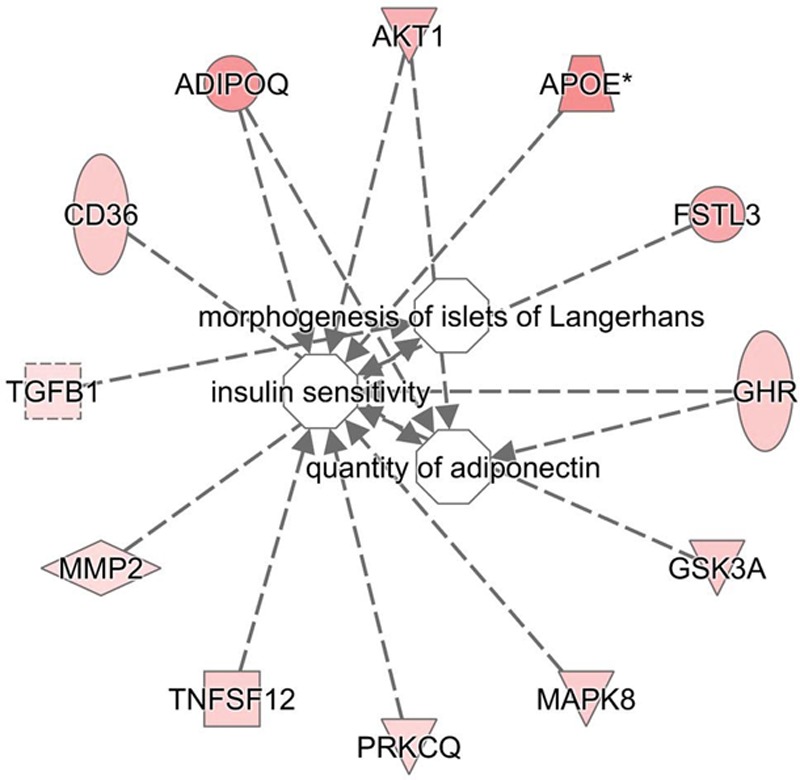
**Proteins significantly altered by torcetrapib associated with insulin sensitivity or pancreatic β-cell function.** Nodes represent gene symbol name, corresponding to protein measured. Degree of intensity of the node color (red) indicates degree of significance of false discovery rate *P* value. Dashed lines correspond to the implicated function of the given proteins according to the Ingenuity Pathway Analysis knowledge bank. Node shapes denote cytokine (□), growth factor (□), kinase (▽), peptidase (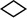), transmembrane receptor (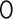), transporter (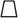), and other (○). ADIPO1 indicates adiponectin; C1Q, and collagen domain containing; APOE, apolipoprotein E, FSTL3, follistatin-like 3; GHR, growth hormone receptor; MAPK8, mitogen-activated protein kinase-8; MMP2, matrix metalloproteinase-2; PRKCQ, protein kinase Cθ type; TGFB1, transforming growth factor-β1; and TNSFSF12, tumor necrosis factor superfamily member 12. *Given protein was represented in the modified aptamer assay more than once.

In the torcetrapib plus atorvastatin arm, of the 986 proteins measured, 200 proteins changed significantly in a univariate analysis at a false discovery rate of 5% (Table II in the online-only Data Supplement). Of the 986 proteins entered into the IPA, Uniprot identification names of 980 were recognized by IPA and used as the total number of background proteins. A pathway analysis of the 200 proteins affected by torcetrapib showed significant enrichment for proteins involved in inflammation and immunity (Table 2). Specifically, among the top 10 IPA canonical pathways (canonical pathways are well-characterized metabolic and cell signaling pathways), 8 are involved in inflammation and immunity (Table 2: B-cell receptor signaling, PPARα/RXRα [peroxisome proliferator-activated receptor-α/retinoid X receptor-α] activation, RAR [retinoic acid receptor] activation, role of NFAT [nuclear factor of activated T cells] in regulation of the immune response, PI3K [phosphoinositide 3-kinase] signaling in B lymphocytes, T-cell receptor signaling, sphingosine-1-phosphate signaling, and triggering receptor expressed on myeloid cells 1 signaling). All the proteins contained within each of these top 10 IPA canonical pathways and those affected by torcetrapib are shown in Figures II through XI in the online-only Data Supplement. In addition, on the basis of IPA, a total of 19 proteins altered by torcetrapib are associated with endocrine system development and function, 8 of which pertain to aldosterone synthesis or function, 9 to insulin sensitivity (ie, glycemic control), and 2 to pancreatic β-cell function (ie, glycemic control; Table 3 and Figures 2 and 3).

**Table 2.**
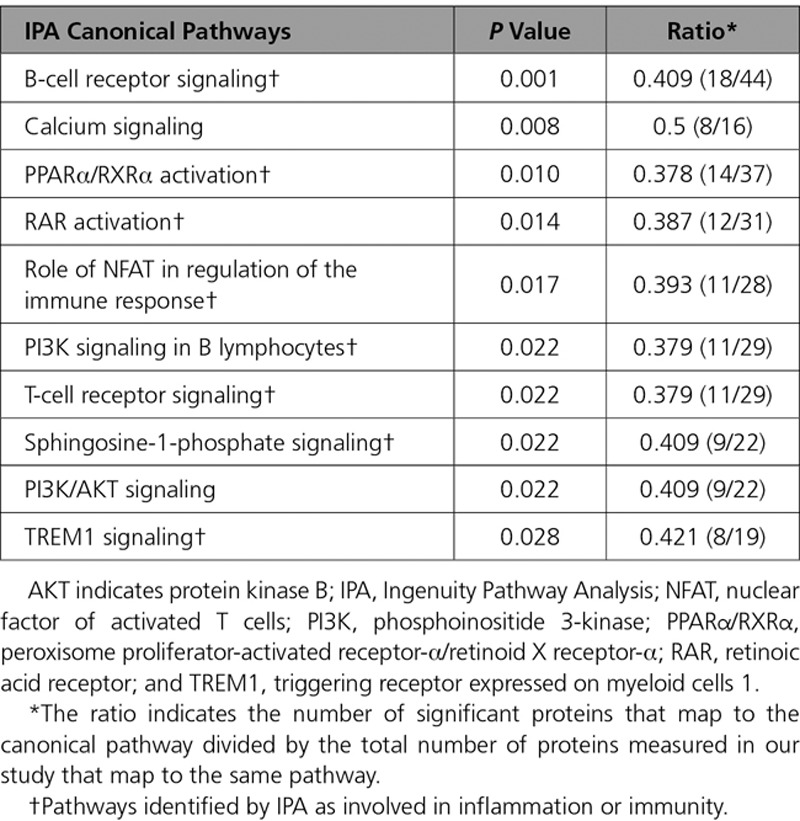
Top 10 IPA Canonical Pathways of the 200 Proteins Significantly Altered by Torcetrapib

**Table 3.**
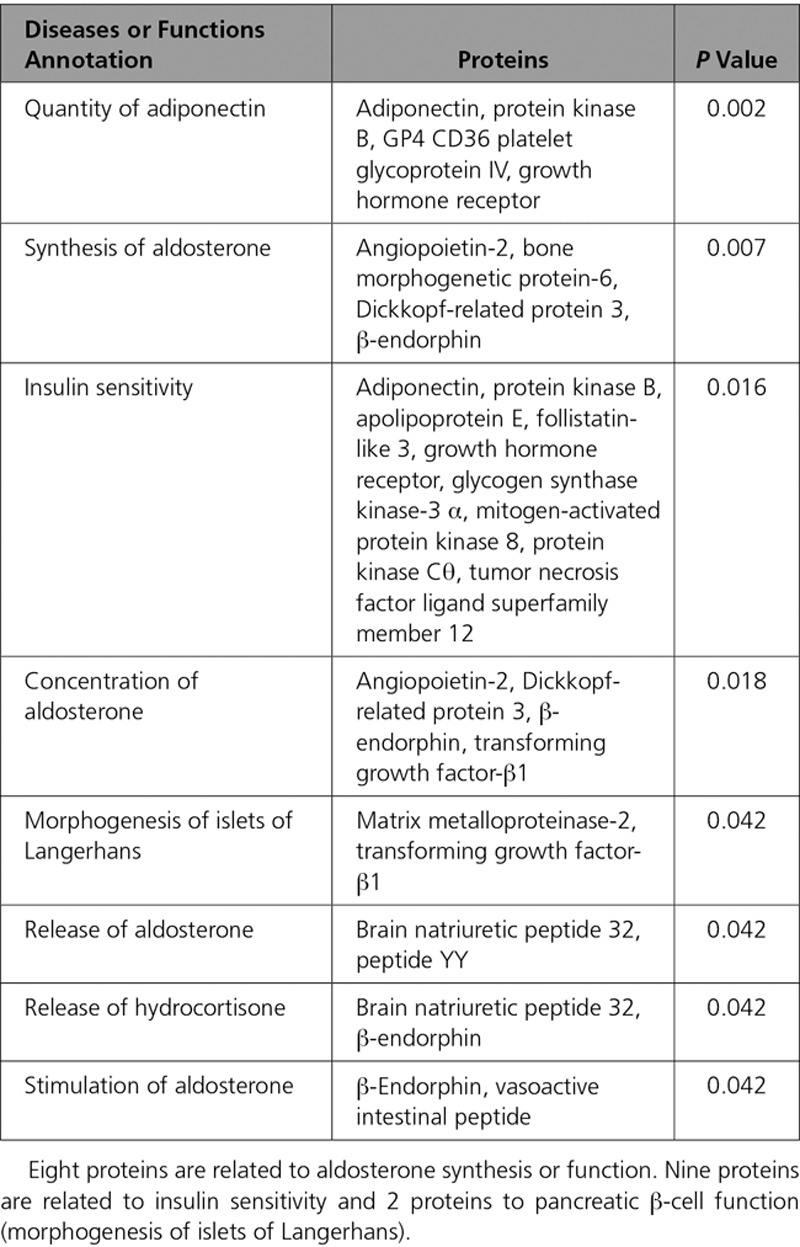
Proteins Significantly Altered by Torcetrapib Associated With Endocrine System Development and Function

To validate our results derived from IPA, we analyzed the 200 proteins changed by torcetrapib using 2 additional pathway analysis resources. Table III in the online-only Data Supplement shows the top 10 pathways identified by Reactome, and Table IV in the online-only Data Supplement shows the top 20 KEGG and BioCarta pathways identified by DAVID. Notably, similar to our results from IPA, we found significant enrichment in pathways involving inflammatory and immune functions.

There were 18 proteins that changed significantly in the atorvastatin-only arm (Table V in the online-only Data Supplement), insufficient for a pathway analysis. Twelve of these 18 proteins changed both in the torcetrapib plus atorvastatin and in the atorvastatin-only arm. Removing the 12 proteins shared by the 2 treatment arms from the 200 proteins in the torcetrapib plus atorvastatin arm had little effect on the IPA top canonical pathway analysis results (Table VI in the online-only Data Supplement).

### Protein Risk Scores at Baseline

At baseline, the 9-protein risk scores were similar among the 2 treatment arms (torcetrapib plus atorvastatin, 7.7%; atorvastatin only, 7.2%; *P*=0.16). Among participants assigned to torcetrapib plus atorvastatin, the baseline 9-protein risk scores were 10.5% and 6.5% for cases and controls, respectively (*P*=0.00046), and for participants assigned to atorvastatin only, the baseline risk scores were 8.44% and 6.0% for cases and controls, respectively (*P*=0.0066).

### Changes in Protein Risk Scores With Treatment

**Figure 4.**
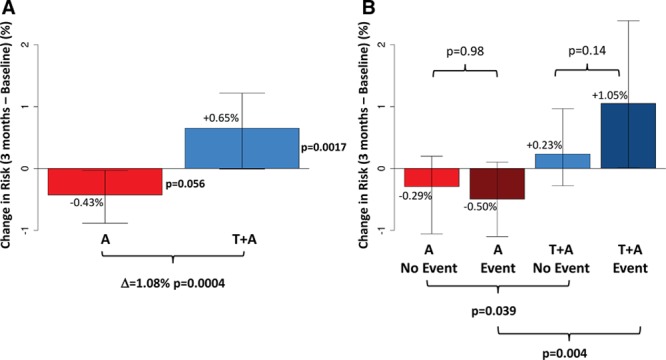
**Within-participant changes in 9-protein risk score, baseline to 3 months. A**, Percent change in risk (3 months minus baseline) by treatment group. **B**, Percent change in risk (3 months minus baseline) by treatment group and event status. Legend to bar charts: Bar height extends to the median change in risk, and the whiskers represent the 95% confidence interval about the median. Labels at the top of each bar are the median risk change. *P* values on the sides of the bars are from testing with a Wilcoxon signed-rank test the null hypothesis that the risk change is distributed symmetrically with a median of zero. *P* values in brackets are from testing with a Wilcoxon rank-sum test the null hypothesis of equal medians for the 2 populations. A indicates atorvastatin; and T, torcetrapib.

Table 4 and Figure 4 show that from baseline to 3 months, treatment with torcetrapib plus atorvastatin was associated with a within-participant increase in the absolute 9-protein risk score of 0.65% (*P*=0.0017) in all participants, 1.05% (*P*=0.006) in the cases, and 0.23% (*P*=0.10) in the controls. Compared across the 2 treatment arms from baseline to 3 months, the 9-protein risk score increased in the torcetrapib plus atorvastatin arm compared with the atorvastatin-only arm in all participants by 1.08% (*P*=0.0004), in participants with events by 1.55% (*P*=0.004), and in participants with no events by 0.52% (*P*=0.039).

**Table 4.**
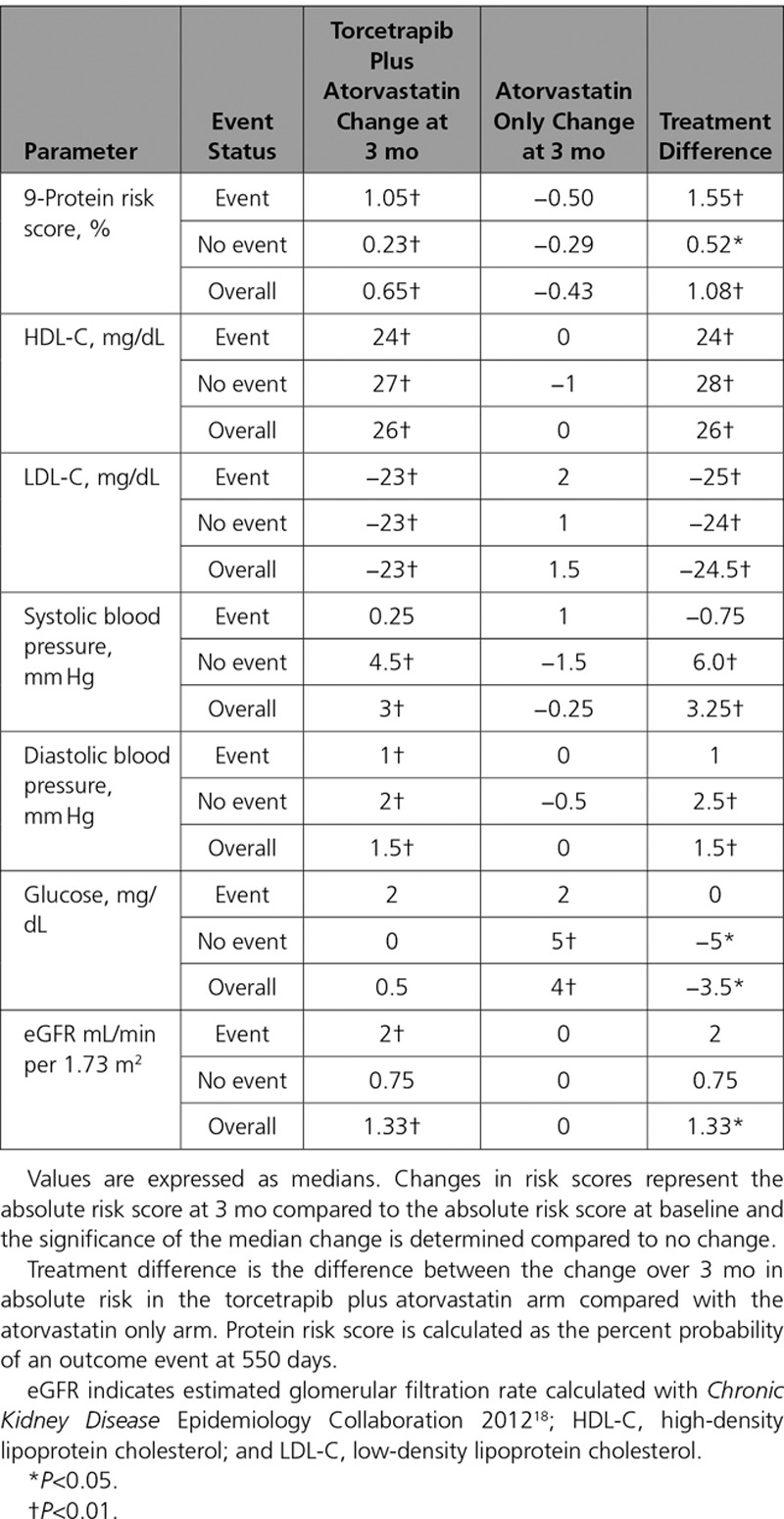
Changes in Key Parameters From Baseline to 3 Months in the 2 Treatment Arms

The changes in 9-protein risk with torcetrapib for the entire population of cases and controls showed a single distribution with no discontinuity (Ansari-Bradley test, *P*=0.15), suggesting that torcetrapib affected all exposed individuals to some extent (Figure XII in the online-only Data Supplement).

### Changes in Framingham Risk Scores With Treatment

Framingham secondary event risk scores^17^ were balanced across the 2 treatment arms at baseline (*P*=0.83). From baseline to 3 months, the Framingham risk score within participants decreased (mean [SD]) from 6.1% (2.5%) to 4.6% (2.0%) in the torcetrapib plus atorvastatin arm (*P*=2.2e−16) and remained unchanged from 6.1% (2.9%) to 6.2% (2.9%) in the atorvastatin alone arm (*P*=0.16). Cross-treatment comparison of the within-participant changes revealed a highly significant reduction in absolute Framingham risk favoring torcetrapib plus atorvastatin over atorvastatin alone (−1.5%; *P*=2e−16).

### Survey of Changes in Proteins Described Previously to Be Prognostic of Cardiovascular Events

We address here 200 proteins previously found to be prognostic of the same cardiovascular outcomes in a population of patients with coronary heart disease similar to that in the present study, measured by the same modified aptamer assay.^8^ In the present study, 49 of those 200 prognostic proteins changed significantly within the torcetrapib arm between baseline and 3 months (false discovery rate–corrected *P*<0.05; Table VII in the online-only Data Supplement). Among these proteins, 37 (76%) changed in the direction of increased risk and 12 proteins changed in the direction of reduced risk. Within the atorvastatin-only arm, only 5 of the 200 prognostic proteins changed significantly (Table VII in the online-only Data Supplement).

## Discussion

In this retrospective proteomic analysis of the ILLUMINATE trial,^1^ a survey of 1129 proteins at baseline and at 3 months of treatment revealed heretofore unknown and widespread effects of torcetrapib on inflammation and immunity, with 8 of the top 10 canonical pathways affected by torcetrapib involved in inflammatory and immune functions. Pathway analysis also reinforced the previous finding of increased aldosterone levels with torcetrapib^1^ by pinpointing 8 plasma proteins altered by torcetrapib that relate to aldosterone synthesis or function. Pathway analysis also provided further explanation for the reported improvement in glycemic control with torcetrapib in the ILLUMINATE trial^19,20^ by identifying 9 proteins related to insulin sensitivity and 2 proteins related to pancreatic β-cell function that are affected by torcetrapib. In addition, a previously validated 9-protein cardiovascular risk score^8^ predicted the harm associated with the use of torcetrapib at just 3 months of treatment that had previously been observed by adverse cardiovascular and mortality outcomes at 550 days (≈18 months) of treatment when the study was halted.^1^ The results of this study suggest that a protein-based risk assessment embedded within a larger proteomic survey may prove to be useful in the clinical and mechanistic evaluation of therapies to prevent harm to patients.

The need for improved assessment of cardiovascular safety reached a turning point in 2004 to 2007 with a series of unfortunate events. First, rofecoxib (Vioxx), a cyclooxygenase-2 inhibitor, was withdrawn from the market because of an adverse cardiovascular safety profile missed during its development.^21,22^ Then, the diabetes drug rosiglitazone (Avandia) was suspected of raising the risk of myocardial infarction, which also was not detected during its development.^23^ Finally, the ILLUMINATE trial results were even more concerning: There was a clear adverse effect of torcetrapib on cardiovascular and all-cause mortality outcomes despite a remarkable improvement in lipid profile.^1^

The harmful effects of drugs could potentially be predicted early in clinical trials by the application of the modern tools of precision medicine. Measurement of proteins in particular is advantageous because they are the key regulators of biological processes in health and in diseases. Unlike genes, proteins can change over time to reflect alterations in disease risk. Although genetic mendelian randomization approaches can also predict the effects of drugs on the intended pharmacological targets,^24^ only proteomic analyses can pinpoint any unsuspected drug toxicities that are related to off-target effects of the drug molecule. Modified aptamers have advanced the field of proteomics by enabling rapid quantification of hundreds or even thousands of proteins in a small volume of blood.^8,10–13^ The technique is highly sensitive, with a median limit of detection ≈1 pg/mL, and precise, with median intra-assay and interassay coefficients of variation <4%. The binding of modified aptamers to their intended protein target is highly specific and quantitative, as shown by the use of mass spectrometry as an orthogonal technique.^12^

The first objective in this study, enabled by a large-scale proteomics survey, was to derive a mechanistic understanding of the biological effects of torcetrapib. The aim was to identify those proteins with levels that were significantly altered by torcetrapib and to organize any such differentially expressed proteins into biological pathways.^25^ Unexpectedly, this process revealed that torcetrapib exerted biological effects that were widespread, altering the plasma concentrations of 200 of the 986 proteins (20.3%) successfully measured (Table II in the online-only Data Supplement). Furthermore, when organized according to biological pathways, this approach revealed that 8 of the top 10 canonical pathways affected by torcetrapib relate to inflammatory and immune functions (Table II and Figures II–XI in the online-only Data Supplement), effects not previously associated with torcetrapib. These results obtained through IPA were reinforced with Reactome (Table III in the online-only Data Supplement) and DAVID (Table IV in the online-only Data Supplement), which similarly revealed significant enrichment in pathways involving inflammation and immunity. This finding not only has important implications for increased cardiovascular risk observed with torcetrapib in the ILLUMINATE trial, because inflammation and immunity are central to cardiovascular disease,^26^ but also supports the possible contribution of the immune surveillance and inflammatory axes to the excess noncardiovascular deaths caused by sepsis and cancer with torcetrapib reported in the ILLUMINATE trial.^1^ Whether these inflammatory and immune function activities of torcetrapib are related specifically to the molecule itself or reflect accumulation of proinflammatory HDL particles from the inhibition of cholesterol ester transfer protein^27^ can be resolved by proteomic investigation of trials that used other agents in the same class but with a different molecular structure. Some of the 200 proteins that were subjected to the IPA pathway analysis in the torcetrapib arm also changed in the atorvastatin-only arm. Excluding all 12 such proteins from the analysis did not materially alter our findings (Table VI in the online-only Data Supplement). Because atorvastatin inhibits cholesterol ester transfer protein to raise HDL cholesterol,^28^ some of these shared proteins may relate to inhibition of the same cholesterol ester transfer protein target by torcetrapib and atorvastatin. Many proteins that we measured in plasma are secretable, evidenced by their gene encoding for a signal sequence or by meeting other established criteria for secretion.^29^ Other proteins we measured are typically considered intracellular; the mechanisms by which they appear in plasma remain largely speculative but include release by cell necrosis,^30^ cell apoptosis,^30^ reversible membrane permeabilization,^30^ formation of cell membrane blebs,^31^ and extracellular vesicles.^31^

Our proteomic pathway analysis showed that torcetrapib in ILLUMINATE also had major endocrine effects, particularly on aldosterone and glycemic control. Aldosterone levels had been measured in a post hoc exploratory analysis to explain the observed elevations in blood pressure, reductions in potassium, and elevation in bicarbonate among patients who received torcetrapib.^1^ At the time of the original publication, the mechanisms by which torcetrapib raised aldosterone levels were unknown. In the present study, IPA identified significant enrichment in 8 proteins involved in aldosterone synthesis, concentration, release, and stimulation (angiopoietin-2, bone morphogenetic protein 6, Dickkopf-related protein 3, β-endorphin/proopiomelanocortin, transforming growth factor-β1, natriuretic peptide [precursor] B, peptide YY, and vasoactive intestinal peptide; Table 3 and Figure 2). Furthermore, the reduction in renin and the absence of an increase in adrenocorticotropic hormone observed in the present proteomic analysis are also consistent with a direct action of torcetrapib leading to hyperaldosteronism. The ILLUMINATE trial also reported improved glycemic control with torcetrapib, explained predominantly by improved insulin sensitivity.^19,20^ Notably, pathway analysis identified 9 proteins related to insulin sensitivity and 2 proteins related to pancreatic β-cell function altered by torcetrapib, providing potential mechanistic leads (Table 3 and Figure 3).

The second objective in the present study was to evaluate whether a previously validated 9-protein cardiovascular risk score^8^ could detect the adverse effects of torcetrapib at 3 months of treatment. The results show that this was achieved; despite differences in the study design and end points used in the present analysis compared with the larger ILLUMINATE trial, the absolute magnitude of the increase in risk of 1.08% predicted by the 9-protein risk score is consistent with the observed absolute 1.2% increase in cardiovascular events with torcetrapib.^1^ This increased risk with torcetrapib likely affected all participants exposed because torcetrapib-associated changes in the 9-protein risk score represent a single statistical distribution (by the Ansari-Bradley test^16^). We used the 9-protein risk score to predict the response of torcetrapib because this risk score was mathematically derived from 1130 protein biomarkers that we previously tested; it is externally validated^8^ and applies to a population of individuals with stable coronary heart disease similar to those in the ILLUMINATE study. We present the 9-protein score as a proof of principle, acknowledging that other protein-based risk scores might perform well. Furthermore, 49 of the 200 proteins that were previously shown to be prognostic of cardiovascular and mortality outcomes in patients with coronary heart disease^8^ were affected by torcetrapib. The observation that 37 of 49 prognostic proteins moved in the direction of adverse risk would create a significant concern for torcetrapib even in the absence of a formal 9-protein risk score. Twelve of 49 prognostic proteins changed in the direction of reduced risk, suggesting that torcetrapib may have had some beneficial effects (perhaps mediated by LDL cholesterol reduction or improved glycemic control) but that these effects were masked by the dominant harmful effects of the drug. Although these 37 prognostic proteins included 2 established cardiovascular biomarkers, notably troponin I (which is part of the 9-protein risk score) and brain (B-type) natriuretic peptide, C-reactive protein did not predict the harm from torcetrapib in this analysis. The observation that treatment-associated changes in C-reactive protein are strongly prognostic of the clinical benefit derived from the cholesterol-lowering agents statins^32–35^ and ezetimibe^36^ but not prognostic of the harm from torcetrapib points to the advantages of a large-scale proteomic screen because individual biomarkers cannot be reasonably expected to cover all relevant biological systems and will inevitably miss some adverse drug effects. The up-front choice of which biomarkers would be affected is a guessing game that is rendered unnecessary by measuring them all (or as many as current technology allows).

The hypertensive effects of torcetrapib were known early in its development,^2^ and one might reasonably question whether they should have raised greater concern. However, a formal calculation of the Framingham secondary risk score in the present study shows convincingly that the benefits of torcetrapib on steeply raising HDL cholesterol and lowering LDL cholesterol would be expected to outweigh the detriment in cardiovascular risk caused by the level of increased blood pressure observed. The improvement in Framingham risk score with torcetrapib clearly represents a false result and illustrates how misleading risk calculation based on traditional risk factors would be in this instance, in contrast to the correct risk prediction based on the plasma proteome. The likely flaw in the Framingham risk calculation is that it focuses on HDL cholesterol levels without accounting for HDL particle function.^5^

### Limitations

The atorvastatin-only treatment arm had a slight, borderline significant reduction in the 9-protein risk score at 3 months from baseline (0.43% absolute risk reduction, *P*=0.056), when perhaps no effect would have been expected. This is likely a “spillover” effect from the atorvastatin dose uptitration during the run-in phase of the study just before treatment randomization.^1^ In this regard, it is known that higher doses of atorvastatin reduce cardiovascular risk more than lower doses.^37^ This potential spillover effect of atorvastatin uptitration would be expected to affect both treatment arms similarly.

Another potential limitation of the study is the nested case-control design of the present study. Available information strongly supports the use of nested case-control designs for the evaluation of risk prediction measures in cardiovascular disease.^14^ Our statistical analyses focused primarily on group changes (ie, the increased risk we predicted approximated the risk observed in the trial), and we did not examine how well our predictions may have performed in individual participants (eg, by discrimination testing). Our results apply only to the population tested. It is not known how our findings would translate to cohorts with other characteristics.

Last, some proteins are shared among ≥2 biological pathways. For the purposes of this study, we assumed that a change in a protein concentration affects all the pathways that contain it. The validity of that assumption will be clarified by future studies.

### Conclusions

The application of proteomics provides strong evidence and potential mechanistic explanations (through immunity, inflammation, and endocrine effects) for harmful biological effects of torcetrapib that could have alerted investigators early in the ILLUMINATE trial or could have been useful when making the decision to proceed to this phase III study. Admittedly, the results of our study are novel and thus will need further validation. For example, it would be interesting to compare these results with drugs of the same class that may not share molecule-specific off-target effects of torcetrapib such as Lilly’s evacetrapib, Roche’s dalcetrapib^38^ (both lacked efficacy in phase III trials but did not cause harm), or Merck’s anacetrapib. More broadly, our proteomic study provides evidence for the recent scientific statement from the American Heart Association that proteomics can have a “transformative impact for cardiovascular health and disease.”^9^

## Acknowledgments

The authors thank the Assay Execution Team at SomaLogic, Inc., for performing the SOMAscan assays; Christina Lee and Jennifer Hajj for providing logistical support; and Fraser Gaspar for statistical advice.

## Sources of Funding

The ILLUMINATE trial was supported by Pfizer Inc., and the proteomic analysis by SomaLogic, Inc. Dr Ganz’s proteomic research is supported by National Institutes of Health grants 1RO1HL129856, 1UO1DK108809, and 1R01AG052964.

## Disclosures

Dr Murthy reports no conflict. Dr Ganz serves on a medical advisory board to SomaLogic, Inc., for which he accepts no salary, honoraria, or any other financial incentives. Drs DeLisle, Ostroff, Weiss, and Williams are employees of SomaLogic, Inc. Drs Hyde and Malarstig are employees of Pfizer Inc.

Pfizer Inc. and SomaLogic, Inc., had a role in the design and conduct of the study; collection, management, analysis, and interpretation of the data; and preparation, review, and approval of the manuscript. Dr Ganz had the ultimate responsibility for all aspects of this study. Pfizer Inc. and SomaLogic, Inc., had no veto rights concerning the decision to submit the manuscript for publication.

## Supplementary Material

## References

[1] Barter PJ, Caulfield M, Eriksson M, Grundy SM, Kastelein JJ, Komajda M, Lopez-Sendon J, Mosca L, Tardif JC, Waters DD, Shear CL, Revkin JH, Buhr KA, Fisher MR, Tall AR, Brewer B, ILLUMINATE Investigators (2007). Effects of torcetrapib in patients at high risk for coronary events.. N Engl J Med.

[2] Tall AR, Yvan-Charvet L, Wang N (2007). The failure of torcetrapib: was it the molecule or the mechanism?. Arterioscler Thromb Vasc Biol.

[3] Berenson A (2006). End of drug trial is a big loser for Pfizer.. New York Times.

[4] Everett BM, Smith RJ, Hiatt WR (2015). Reducing LDL with PCSK9 inhibitors: the clinical benefit of lipid drugs.. N Engl J Med.

[5] Vickers KC, Remaley AT (2014). HDL and cholesterol: life after the divorce?. J Lipid Res.

[6] Robb MA, McInnes PM, Califf RM (2016). Biomarkers and surrogate endpoints: developing common terminology and definitions.. JAMA.

[7] Brody E, Gold L, Mehan M, Ostroff R, Rohloff J, Walker J, Zichi D (2012). Life’s simple measures: unlocking the proteome.. J Mol Biol.

[8] Ganz P, Heidecker B, Hveem K, Jonasson C, Kato S, Segal MR, Sterling DG, Williams SA (2016). Development and validation of a protein-based risk score for cardiovascular outcomes among patients with stable coronary heart disease.. JAMA.

[9] Lindsey ML, Mayr M, Gomes AV, Delles C, Arrell DK, Murphy AM, Lange RA, Costello CE, Jin YF, Laskowitz DT, Sam F, Terzic A, Van Eyk J, Srinivas PR, American Heart Association Council on Functional Genomics and Translational Biology, Council on Cardiovascular Disease in the Young, Council on Clinical Cardiology, Council on Cardiovascular and Stroke Nursing, Council on Hypertension, and Stroke Council (2015). Transformative impact of proteomics on cardiovascular health and disease: a scientific statement from the American Heart Association.. Circulation.

[10] Sabatine MS (2016). Using aptamer-based technology to probe the plasma proteome for cardiovascular disease prediction.. JAMA.

[11] Gold L, Ayers D, Bertino J, Bock C, Bock A, Brody EN, Carter J, Dalby AB, Eaton BE, Fitzwater T, Flather D, Forbes A, Foreman T, Fowler C, Gawande B, Goss M, Gunn M, Gupta S, Halladay D, Heil J, Heilig J, Hicke B, Husar G, Janjic N, Jarvis T, Jennings S, Katilius E, Keeney TR, Kim N, Koch TH, Kraemer S, Kroiss L, Le N, Levine D, Lindsey W, Lollo B, Mayfield W, Mehan M, Mehler R, Nelson SK, Nelson M, Nieuwlandt D, Nikrad M, Ochsner U, Ostroff RM, Otis M, Parker T, Pietrasiewicz S, Resnicow DI, Rohloff J, Sanders G, Sattin S, Schneider D, Singer B, Stanton M, Sterkel A, Stewart A, Stratford S, Vaught JD, Vrkljan M, Walker JJ, Watrobka M, Waugh S, Weiss A, Wilcox SK, Wolfson A, Wolk SK, Zhang C, Zichi D (2010). Aptamer-based multiplexed proteomic technology for biomarker discovery.. PLoS One.

[12] Ngo D, Sinha S, Shen D, Kuhn EW, Keyes MJ, Shi X, Benson MD, O’Sullivan JF, Keshishian H, Farrell LA, Fifer MA, Vasan RS, Sabatine MS, Larson MG, Carr SA, Wang TJ, Gerszten RE (2016). Aptamer-based proteomic profiling reveals novel candidate biomarkers and pathways in cardiovascular disease.. Circulation.

[13] Gramolini A, Lau E, Liu PP (2016). Identifying low-abundance biomarkers: aptamer-based proteomics potentially enables more sensitive detection in cardiovascular diseases.. Circulation.

[14] Ganna A, Reilly M, de Faire U, Pedersen N, Magnusson P, Ingelsson E (2012). Risk prediction measures for case-cohort and nested case-control designs: an application to cardiovascular disease.. Am J Epidemiol.

[15] Armbruster DA, Pry T (2008). Limit of blank, limit of detection and limit of quantitation.. Clin Biochem Rev.

[16] Ansari AR, Bradley RA (1960). Rank-sum tests for dispersions.. Ann Math Statist.

[17] D’Agostino RB, Russell MW, Huse DM, Ellison RC, Silbershatz H, Wilson PW, Hartz SC (2000). Primary and subsequent coronary risk appraisal: new results from the Framingham study.. Am Heart J.

[18] Inker LA, Schmid CH, Tighiouart H, Eckfeldt JH, Feldman HI, Greene T, Kusek JW, Manzi J, Van Lente F, Zhang YL, Coresh J, Levey AS, CKD-EPI Investigators (2012). Estimating glomerular filtration rate from serum creatinine and cystatin C.. N Engl J Med.

[19] Barter PJ, Rye KA, Tardif JC, Waters DD, Boekholdt SM, Breazna A, Kastelein JJ (2011). Effect of torcetrapib on glucose, insulin, and hemoglobin A1c in subjects in the Investigation of Lipid Level Management to Understand its Impact in Atherosclerotic Events (ILLUMINATE) trial.. Circulation.

[20] Wiviott SD (2011). ILLUMINATE sheds more light.. Circulation.

[21] Waxman HA (2005). The lessons of Vioxx: drug safety and sales.. N Engl J Med.

[22] Mukherjee D, Nissen SE, Topol EJ (2001). Risk of cardiovascular events associated with selective COX-2 inhibitors.. JAMA.

[23] Nissen SE, Wolski K (2007). Effect of rosiglitazone on the risk of myocardial infarction and death from cardiovascular causes.. N Engl J Med.

[24] Thanassoulis G, O’Donnell CJ (2009). Mendelian randomization: nature’s randomized trial in the post-genome era.. JAMA.

[25] Khatri P, Sirota M, Butte AJ (2012). Ten years of pathway analysis: current approaches and outstanding challenges.. PLoS Comput Biol.

[26] Libby P, Hansson GK (2015). Inflammation and immunity in diseases of the arterial tree: players and layers.. Circ Res.

[27] Smith JD (2010). Dysfunctional HDL as a diagnostic and therapeutic target.. Arterioscler Thromb Vasc Biol.

[28] Chapman MJ, Le Goff W, Guerin M, Kontush A (2010). Cholesteryl ester transfer protein: at the heart of the action of lipid-modulating therapy with statins, fibrates, niacin, and cholesteryl ester transfer protein inhibitors.. Eur Heart J.

[29] Lin H, Lee E, Hestir K, Leo C, Huang M, Bosch E, Halenbeck R, Wu G, Zhou A, Behrens D, Hollenbaugh D, Linnemann T, Qin M, Wong J, Chu K, Doberstein SK, Williams LT (2008). Discovery of a cytokine and its receptor by functional screening of the extracellular proteome.. Science.

[30] Amgalan D, Pekson R, Kitsis RN (2017). Troponin release following brief myocardial ischemia: apoptosis versus necrosis.. J Am Coll Cardiol Basic Trans Science.

[31] Hickman PE, Potter JM, Aroney C, Koerbin G, Southcott E, Wu AH, Roberts MS (2010). Cardiac troponin may be released by ischemia alone, without necrosis.. Clin Chim Acta.

[32] Ridker PM, Danielson E, Fonseca FA, Genest J, Gotto AM, Kastelein JJ, Koenig W, Libby P, Lorenzatti AJ, Macfadyen JG, Nordestgaard BG, Shepherd J, Willerson JT, Glynn RJ, JUPITER Trial Study Group (2009). Reduction in C-reactive protein and LDL cholesterol and cardiovascular event rates after initiation of rosuvastatin: a prospective study of the JUPITER trial.. Lancet.

[33] Morrow DA, de Lemos JA, Sabatine MS, Wiviott SD, Blazing MA, Shui A, Rifai N, Califf RM, Braunwald E (2006). Clinical relevance of C-reactive protein during follow-up of patients with acute coronary syndromes in the Aggrastat-to-Zocor Trial.. Circulation.

[34] Nissen SE, Tuzcu EM, Schoenhagen P, Crowe T, Sasiela WJ, Tsai J, Orazem J, Magorien RD, O’Shaughnessy C, Ganz P, Reversal of Atherosclerosis with Aggressive Lipid Lowering (REVERSAL) Investigators (2005). Statin therapy, LDL cholesterol, C-reactive protein, and coronary artery disease.. N Engl J Med.

[35] Ridker PM, Cannon CP, Morrow D, Rifai N, Rose LM, McCabe CH, Pfeffer MA, Braunwald E, Pravastatin or Atorvastatin Evaluation and Infection Therapy-Thrombolysis in Myocardial Infarction 22 (PROVE IT-TIMI 22) Investigators (2005). C-reactive protein levels and outcomes after statin therapy.. N Engl J Med.

[36] Bohula EA, Giugliano RP, Cannon CP, Zhou J, Murphy SA, White JA, Tershakovec AM, Blazing MA, Braunwald E (2015). Achievement of dual low-density lipoprotein cholesterol and high-sensitivity C-reactive protein targets more frequent with the addition of ezetimibe to simvastatin and associated with better outcomes in IMPROVE-IT.. Circulation.

[37] LaRosa JC, Grundy SM, Waters DD, Shear C, Barter P, Fruchart JC, Gotto AM, Greten H, Kastelein JJ, Shepherd J, Wenger NK, Treating to New Targets (TNT) Investigators (2005). Intensive lipid lowering with atorvastatin in patients with stable coronary disease.. N Engl J Med.

[38] Schwartz GG, Olsson AG, Abt M, Ballantyne CM, Barter PJ, Brumm J, Chaitman BR, Holme IM, Kallend D, Leiter LA, Leitersdorf E, McMurray JJ, Mundl H, Nicholls SJ, Shah PK, Tardif JC, Wright RS, dal-OUTCOMES Investigators (2012). Effects of dalcetrapib in patients with a recent acute coronary syndrome.. N Engl J Med.

