# Supplementary material for: Improving Assessment of Drug Safety Through Proteomics: Early Detection and Mechanistic Characterization of the Unforeseen Harmful Effects of Torcetrapib
Source: Circulation. 2018 Mar 5;137(10):999–1010. doi: 10.1161/CIRCULATIONAHA.117.028213 (PMC5839936; doi:10.1161/CIRCULATIONAHA.117.028213)
Supplement: Supplementary file 1 [file cir-137-0999-s001.pdf]

## SUPPLEMENTAL MATERIAL

**Improving Assessment of Drug Safety through Proteomics: Early Detection and Mechanistic Characterization of the Unforeseen Harmful Effects of Torcetrapib**

Stephen A. Williams, MD, PhD[1*], Ashwin C. Murthy, MD[2*], Robert K. DeLisle, PhD[1], Craig Hyde, PhD[3], Anders Malarstig PhD[4], Rachel Ostroff, PhD[1], Sophie J. Weiss, PhD[1], Mark R. Segal, PhD[5], Peter Ganz, MD[2,6]

**Affiliations:**

[1] SomaLogic Inc. Boulder, Colorado, USA

[2] Department of Medicine, University of California, San Francisco, CA, USA

[3] Pfizer, Worldwide Research and Development, Groton, CT, USA

[4] Pfizer, Worldwide Research and Development, Stockholm, Sweden

[5] Department of Epidemiology and Biostatistics, University of California, San Francisco, CA, USA

[6] Division of Cardiology, Zuckerberg San Francisco General Hospital, San Francisco, CA, USA

* Contributed equally to this work

**TABLE OF CONTENTS**

**Supplemental Method I: The reasons for exclusion of some aptamers/proteins in the ILLUMINATE analysis.** page 4

**Supplemental Method II: 60 bridging samples used as protein standards to align the fluorescence readouts of the same proteins in the same samples across the different assay versions.** page 4

**Supplemental Table I: The distribution of coefficients of variations (%CVs) for all 1129 protein targets in EDTA plasma and %CVs for proteins in the 9-protein model.** page 6

**Supplemental Table II: Proteins that changed significantly in a univariate analysis at 3 months compared to baseline in the torcetrapib plus atorvastatin treatment arm.** pp. 7-13

**Supplemental Table III: Top 10 pathways among the 200 proteins significantly altered by torcetrapib identified by Reactome Pathway Database.** page 14

**Supplemental Table IV: Top 20 KEGG and BIOCARTA Pathways of the 200 proteins significantly altered by torcetrapib identified by DAVID**. pp. 15-16

**Supplemental Table V: Proteins that changed significantly in a univariate analysis at 3 months compared to baseline for the atorvastatin only treatment arm.** page 17

**Supplemental Table VI: The top Ingenuity Pathway Analysis (IPA) 'Canonical Pathways' of the proteins significantly altered by torcetrapib plus atorvastatin with removal of the proteins significantly altered by atorvastatin only.** page 18

**Supplemental Table VII: Proteins previously shown to be prognostic of cardiovascular events that changed between baseline and 3 months.** pp. 19-22

**Supplemental Figure I: Comparison of risk score distributions in participants with known coronary heart disease with risk score distribution in all participants (including diabetic participants enrolled in the study without known CHD).** page 23

**Supplemental Figure II: B cell receptor signaling canonical pathway from Ingenuity Pathway Analysis.** pp. 24-25

**Supplemental Figure III: Calcium signaling canonical pathway from Ingenuity Pathway Analysis.** pp. 26-27

**Supplemental Figure IV: PPARα/RXRα activation canonical pathway from Ingenuity Pathway Analysis.** page 28

**Supplemental Figure V: RAR activation canonical pathway from Ingenuity Pathway Analysis.** pp. 29-30

**Supplemental Figure VI: Role of NFAT in regulation of the immune response canonical pathway from Ingenuity Pathway Analysis.** pp. 31-32

**Supplemental Figure VII: PI3K signaling in B lymphocytes canonical pathway from Ingenuity Pathway Analysis.** pp. 33-34

**Supplemental Figure VIII: T cell receptor signaling canonical pathway from Ingenuity Pathway Analysis.** pp. 35-36

**Supplemental Figure IX: Sphingosine-1-phosphate signaling canonical pathway from Ingenuity Pathway Analysis.** page 37.

**Supplemental Figure X: PI3K/AKT signaling canonical pathway from Ingenuity Pathway Analysis.** page 38.

**Supplemental Figure XI: TREM1 signaling canonical pathway from Ingenuity Pathway Analysis.** pp. 39-40

**Supplemental Figure XII: Cumulative distribution function for within-participants change in risk for the T+A group and the A group showing a small but relatively consistent upward shift in risk in the T+A group for the majority of the study population.** page 41.

**Supplemental References.** page 42.

**<u>Supplemental Method I</u>**: **<u>The reasons for exclusion of some aptamers/proteins in the ILLUMINATE analysis.</u>** The reproducibility experiment of Supplemental Table 1 not only assesses measurement %CV over replicate plasma samples, but also establishes a calibration reference from the calibrator samples. In Illuminate and case/control and cohort studies, each sample represents a different individual or time point etc., not replicates. Therefore, %CV is not used for exclusion/acceptance of protein targets. Instead, five calibrator sample replicates (of the same lot as in Supplemental Table 1) are run on each plate, and then compared to the calibrator reference derived from the reproducibility study, for both a measure of run-to-run variability and assay drift. After normalization, and for each protein target, the ratio of the calibrator reference value to the median value in the five calibrator samples is computed for each plate. If a ratio is outside of 0.6-1.4, that protein target is removed from analysis.

**<u>Supplemental Method II</u>**: **<u>60 bridging samples used as protein standards to align the fluorescence readouts of the same proteins in the same samples across the different assay versions</u>.** Sixty samples from the HUNT3 cohort previously used to validate the 9-protein risk score (the JAMA score), (Ganz et al, JAMA 2016; 315: 2532-2541), were also run in the current Illuminate study. Samples were chosen to represent the full distribution of 9-protein levels in the original study as closely as possible. A robust linear model was fit to the values from the 60 HUNT3 samples run both in the current Illuminate study and in the previous study. This robust linear model was used to map the newer Illuminate assay version back to the older assay version. Even without using the 60 HUNT3 samples to bridge assay versions, but instead bridging by using the JAMA calibrator reference, results were consistent. For example, the table below shows what the p-values would be in Figure 4 of the main text with assay bridging and using the JAMA calibrator reference:

| Comparison | 60 HUNT3 samples bridging | JAMA calibrator reference |
|---|---|---|
| A no event v. A event | 0.98 | 0.97 |
| A no event v. T+A event | 0.039 | 0.035 |
| A event v. T+A event | 0.004 | 0.004 |
| T+A no event v. T+A event | 0.14 | 0.16 |

**Supplemental Table I: The distribution of coefficients of variations (%CVs) for all 1129 protein targets in EDTA plasma and %CVs for proteins in the 9-protein model.**

| % of protein targets | Intra-Assay %CV | Inter-Assay %CV | Total %CV | CVs of 9-protein targets |
|---|---|---|---|---|
| 5% | ≤ 1.4% | ≤ 1.4% | ≤ 2.4% | |
| 25% | ≤ 1.8% | ≤ 2.5% | ≤ 3.1% | MMP12 |
| 50% | ≤ 2.3% | ≤ 2.9% | ≤ 3.9% | SERPINA3, TNNI3, ANGPTL4, SERPINF2, GDF-11/8 |
| 75% | ≤ 3.1% | ≤ 3.8% | ≤ 5.0% | CCL18, C7, ANGPT2 |
| 95% | ≤ 5.7% | ≤ 8.4% | ≤ 10.5% | |

Shown is a summary of the average intra-assay, inter-assay, and total %CVs for 1129 proteins measured by modified aptamers in EDTA plasma across 3 runs. The average was computed from three individual samples performed in triplicate on each run. Reproducibility was assessed by running 16 samples in each of 3 independent assay runs. Three plasma samples were run in triplicate, with five replicates of the associated calibrator sample, and two buffer control wells on each run. The separate runs utilized different operators to simulate typical run-to-run variability. The total %CVs for each protein target was computed across the nine replicate plasma samples over the three plate runs after normalization and calibration. The overall %CVs for intra-assay measurements are generally less than those for inter-assay measurement.

**Supplemental Table II: Proteins that changed significantly in a univariate analysis at 3 months compared to baseline in the torcetrapib plus atorvastatin treatment arm,** truncated at a false discovery rate adjusted p-value <0.05.

| Protein | Entrez Gene Symbol | UniProt ID | log2-Fold Change | FDR p-value |
|---|---|---|---|---|
| Carbonic anhydrase 6 | CA6 | P23280 | 0.304 | 8.88E-23 |
| Renin | REN | P00797 | -0.316 | 8.65E-13 |
| CD209 antigen | CD209 | Q9NNX6 | 0.145 | 5.77E-12 |
| Beta-endorphin | POMC | P01189 | 0.131 | 7.50E-10 |
| Apolipoprotein A-I | APOA1 | P02647 | -0.116 | 2.18E-09 |
| Serum amyloid A-1 protein | SAA1 | P0DJI8 | 0.260 | 2.78E-06 |
| Interleukin-11 receptor subunit alpha | IL11RA | Q14626 | 0.153 | 2.78E-06 |
| Neurogenic locus notch homolog protein 3 | NOTCH3 | Q9UM47 | 0.103 | 3.15E-06 |
| 3-hydroxyacyl-CoA dehydrogenase type-2 | HSD17B10 | Q99714 | -0.364 | 1.19E-05 |
| Thrombospondin-2 | THBS2 | P35442 | 0.143 | 1.95E-05 |
| Apolipoprotein E | APOE | P02649 | -0.159 | 1.96E-05 |
| Troponin I; cardiac muscle | TNNI3 | P19429 | 0.118 | 3.01E-05 |
| Apolipoprotein E (isoform E4) | APOE | P02649 | -0.127 | 3.92E-05 |
| Adiponectin | ADIPOQ | Q15848 | 0.084 | 4.54E-05 |
| Dickkopf-related protein 3 | DKK3 | Q9UBP4 | 0.077 | 1.18E-04 |
| Protein disulfide-isomerase A3 | PDIA3 | P30101 | -0.139 | 1.31E-04 |
| Carbohydrate sulfotransferase 15 | CHST15 | Q7LFX5 | 0.058 | 1.89E-04 |
| Apolipoprotein E (isoform E3) | APOE | P02649 | -0.087 | 2.26E-04 |
| Follistatin-related protein 3 | FSTL3 | O95633 | 0.058 | 2.79E-04 |
| Dual 3';5'-cyclic-AMP and -GMP phosphodiesterase 11A | PDE11A | Q9HCR9 | -0.123 | 2.79E-04 |
| Advanced glycosylation end product-specific receptor; soluble | AGER | Q15109 | 0.092 | 2.79E-04 |
| Platelet-activating factor acetylhydrolase | PLA2G7 | Q13093 | -0.062 | 2.97E-04 |
| Granulins | GRN | P28799 | 0.046 | 4.46E-04 |
| Cadherin-2 | CDH2 | P19022 | 0.042 | 4.46E-04 |
| Allograft inflammatory factor 1 | AIF1 | P55008 | -0.085 | 4.89E-04 |
| RAC-alpha/beta/gamma serine/threonine-protein kinase | AKT1 AKT2 AKT3 | P31749 P31751 Q9Y243 | -0.160 | 6.30E-04 |
| Bone morphogenetic protein 6 | BMP6 | P22004 | 0.057 | 6.30E-04 |
| Fetuin-B | FETUB | Q9UGM5 | 0.046 | 9.08E-04 |
| Metalloproteinase inhibitor 2 | TIMP2 | P16035 | 0.077 | 9.08E-04 |
| Alpha-2-antiplasmin | SERPINF2 | P08697 | -0.047 | 9.27E-04 |
| MAP kinase-activated protein kinase 3 | MAPKAPK3 | Q16644 | -0.112 | 9.48E-04 |

| Protein | Entrez Gene Symbol | UniProt ID | log2-Fold Change | FDR p-value |
|---|---|---|---|---|
| Dynein light chain roadblock-type 1 | DYNLRB1 | Q9NP97 | -0.206 | 9.48E-04 |
| Endothelial cell-specific molecule 1 | ESM1 | Q9NQ30 | 0.073 | 9.48E-04 |
| Spondin-1 | SPON1 | Q9HCB6 | 0.063 | 1.24E-03 |
| Aflatoxin B1 aldehyde reductase member 2 | AKR7A2 | O43488 | -0.268 | 1.34E-03 |
| Ras-related C3 botulinum toxin substrate 1 | RAC1 | P63000 | -0.227 | 1.35E-03 |
| Receptor-type tyrosine-protein kinase FLT3 | FLT3 | P36888 | -0.150 | 1.43E-03 |
| Tyrosine-protein kinase Lyn | LYN | P07948 | -0.362 | 1.46E-03 |
| Sonic hedgehog protein | SHH | Q15465 | 0.197 | 1.46E-03 |
| Calcineurin | PPP3CA PPP3R1 | Q08209 P63098 | -0.136 | 1.46E-03 |
| MAP kinase-activated protein kinase 2 | MAPKAPK2 | P49137 | -0.187 | 1.49E-03 |
| Serine/threonine-protein kinase PAK 6 | PAK6 | Q9NQU5 | -0.352 | 1.59E-03 |
| Tyrosine-protein kinase CSK | CSK | P41240 | -0.246 | 1.83E-03 |
| Beta-Ala-His dipeptidase | CNDP1 | Q96KN2 | -0.082 | 1.96E-03 |
| Copine-1 | CPNE1 | Q99829 | -0.262 | 1.98E-03 |
| Peptidyl-prolyl cis-trans isomerase D | PPID | Q08752 | -0.374 | 1.98E-03 |
| Mitogen-activated protein kinase 1 | MAPK1 | P28482 | -0.204 | 2.03E-03 |
| Tyrosine-protein kinase BTK | BTK | Q06187 | -0.234 | 2.03E-03 |
| Platelet glycoprotein Ib alpha chain | GP1BA | P07359 | -0.046 | 2.05E-03 |
| Mitogen-activated protein kinase 3 | MAPK3 | P27361 | -0.223 | 2.30E-03 |
| Angiopoietin-2 | ANGPT2 | O15123 | 0.068 | 2.30E-03 |
| Apolipoprotein E (isoform E2) | APOE | P02649 | -0.054 | 2.70E-03 |
| Stabilin-2 | STAB2 | Q8WWQ8 | -0.038 | 2.74E-03 |
| Cytosolic non-specific dipeptidase | CNDP2 | Q96KP4 | -0.053 | 2.75E-03 |
| P-selectin | SELP | P16109 | -0.064 | 2.76E-03 |
| Interleukin-8 | CXCL8 | P10145 | 0.057 | 2.95E-03 |
| 14-3-3 protein family | YWHAB;YWHAE;YWHAG;YWHAH;YWHAQ;YWHAZ;SFN | P31946; P62258; P61981; Q04917; P27348; P63104; P31947 | -0.168 | 3.15E-03 |
| 40S ribosomal protein S3 | RPS3 | P23396 | -0.125 | 3.54E-03 |
| Mannose-binding protein C | MBL2 | P11226 | 0.067 | 3.93E-03 |
| Interleukin-13 receptor subunit alpha-1 | IL13RA1 | P78552 | 0.060 | 3.93E-03 |
| Connective tissue growth factor | CTGF | P29279 | -0.074 | 3.97E-03 |
| Kynureninase | KYNU | Q16719 | -0.090 | 4.11E-03 |
| Heterogeneous nuclear ribonucleoprotein A/B | HNRNPAB | Q99729 | -0.118 | 4.21E-03 |
| Thrombopoietin Receptor | MPL | P40238 | -0.056 | 4.33E-03 |

| Protein | Entrez Gene Symbol | UniProt ID | log2-Fold Change | FDR p-value |
|---|---|---|---|---|
| Heat shock 70 kDa protein 1A | HSPA1A | P0DMV8 | 0.080 | 4.40E-03 |
| Calcium/calmodulin-dependent protein kinase type 1 | CAMK1 | Q14012 | -0.046 | 4.42E-03 |
| Integrin alpha-V: beta-5 complex | ITGAV ITGB5 | P06756; P18084 | 0.086 | 4.42E-03 |
| Pyruvate kinase PKM | PKM2 | P14618 | -0.325 | 4.60E-03 |
| 60 kDa heat shock protein; mitochondrial | HSPD1 | P10809 | -0.329 | 4.89E-03 |
| Glycogen synthase kinase-3 alpha/beta | GSK3A GSK3B | P49840 P49841 | -0.227 | 5.81E-03 |
| Persephin | PSPN | O60542 | -0.050 | 5.82E-03 |
| Collagen alpha-1(XXIII) chain | COL23A1 | Q86Y22 | -0.098 | 5.82E-03 |
| Protein kinase C beta type (splice variant beta-II) | PRKCB | P05771 | -0.352 | 5.82E-03 |
| C-type lectin domain family 1 member B | CLEC1B | Q9P126 | -0.113 | 5.97E-03 |
| Interleukin-1 receptor-like 1 | IL1RL1 | Q01638 | 0.060 | 6.11E-03 |
| E-selectin | SELE | P16581 | 0.057 | 6.42E-03 |
| Tyrosine-protein kinase Lyn; isoform B | LYN | P07948 | -0.302 | 6.42E-03 |
| Ribosome maturation protein SBDS | SBDS | Q9Y3A5 | -0.206 | 6.66E-03 |
| Intercellular adhesion molecule 1 | ICAM1 | P05362 | 0.030 | 6.87E-03 |
| Hepcidin | HAMP | P81172 | -0.178 | 6.87E-03 |
| Platelet glycoprotein 4 | CD36 | P16671 | 0.095 | 6.87E-03 |
| Hepatitis A virus cellular receptor 2 | HAVCR2 | Q8TDQ0 | 0.057 | 6.89E-03 |
| Parathyroid hormone-related protein | PTHLH | P12272 | -0.055 | 7.01E-03 |
| Nicotinamide phosphoribosyltransferase | NAMPT | P43490 | -0.167 | 7.12E-03 |
| Interleukin-7 receptor subunit alpha | IL7R | P16871 | 0.063 | 7.30E-03 |
| Growth hormone receptor | GHR | P10912 | -0.050 | 7.30E-03 |
| Alpha-soluble NSF attachment protein | NAPA | P54920 | -0.142 | 7.30E-03 |
| Growth/differentiation factor 2 | GDF2 | Q9UK05 | 0.054 | 7.36E-03 |
| Plasma serine protease inhibitor | SERPINA5 | P05154 | -0.057 | 7.37E-03 |
| Cytochrome P450 3A4 | CYP3A4 | P08684 | -0.107 | 7.55E-03 |
| AMP Kinase (alpha2beta2gamma1) | PRKAA2 PRKAB2 PRKAG1 | P54646 O43741 P54619 | -0.123 | 7.93E-03 |
| Mitogen-activated protein kinase 8 | MAPK8 | P45983 | -0.131 | 7.93E-03 |
| Chymase | CMA1 | P23946 | -0.040 | 8.34E-03 |
| Insulin-like growth factor-binding protein 2 | IGFBP2 | P18065 | 0.099 | 8.40E-03 |
| Mast/stem cell growth factor receptor Kit | KIT | P10721 | -0.051 | 8.42E-03 |
| Thyroxine-binding globulin | SERPINA7 | P05543 | 0.025 | 8.70E-03 |
| SPARC-like protein 1 | SPARCL1 | Q14515 | 0.047 | 8.70E-03 |
| Reticulon-4 receptor | RTN4R | Q9BZR6 | -0.036 | 8.99E-03 |
| Myeloperoxidase | MPO | P05164 | 0.055 | 9.22E-03 |

| Protein | Entrez Gene Symbol | UniProt ID | log2-Fold Change | FDR p-value |
|---|---|---|---|---|
| Vasoactive Intestinal Peptide | VIP | P01282 | -0.068 | 9.75E-03 |
| Inosine-5'-monophosphate dehydrogenase 1 | IMPDH1 | P20839 | -0.174 | 1.00E-02 |
| Methionine aminopeptidase 1 | METAP1 | P53582 | -0.124 | 1.00E-02 |
| Carbonic anhydrase 9 | CA9 | Q16790 | 0.116 | 1.00E-02 |
| T-cell surface glycoprotein CD4 | CD4 | P01730 | -0.048 | 1.00E-02 |
| Cyclin-dependent kinase 8:Cyclin-C complex | CDK8 CCNC | P49336 P24863 | -0.045 | 1.00E-02 |
| Ectonucleoside triphosphate diphosphohydrolase 3 | ENTPD3 | O75355 | -0.034 | 1.07E-02 |
| Tumor necrosis factor ligand superfamily member 12 | TNFSF12 | O43508 | -0.035 | 1.10E-02 |
| Sphingosine kinase 1 | SPHK1 | Q9NYA1 | -0.206 | 1.13E-02 |
| High mobility group protein B1 | HMGB1 | P09429 | -0.138 | 1.16E-02 |
| Pappalysin-1 | PAPPA | Q13219 | -0.048 | 1.16E-02 |
| Insulin-like growth factor-binding protein 6 | IGFBP6 | P24592 | -0.042 | 1.23E-02 |
| Roundabout homolog 2 | ROBO2 | Q9HCK4 | 0.039 | 1.23E-02 |
| Chloride intracellular channel protein 1 | CLIC1 | O00299 | -0.155 | 1.24E-02 |
| Interleukin-24 | IL24 | Q13007 | -0.074 | 1.28E-02 |
| 6-phosphogluconate dehydrogenase; decarboxylating | PGD | P52209 | -0.307 | 1.30E-02 |
| alpha-2-macroglobulin receptor-associated protein | LRPAP1 | P30533 | -0.039 | 1.31E-02 |
| Kallikrein-14 | KLK14 | Q9P0G3 | -0.057 | 1.31E-02 |
| cAMP-regulated phosphoprotein 19 | ARPP19 | P56211 | -0.118 | 1.32E-02 |
| Intercellular adhesion molecule 3 | ICAM3 | P32942 | -0.053 | 1.32E-02 |
| C-type mannose receptor 2 | MRC2 | Q9UBG0 | 0.060 | 1.32E-02 |
| C-C motif chemokine 18 | CCL18 | P55774 | -0.069 | 1.36E-02 |
| Sphingosine kinase 2 | SPHK2 | Q9NRA0 | -0.042 | 1.41E-02 |
| PIK3CA/PIK3R1 | PIK3CA PIK3R1 | P42336 P27986 | -0.071 | 1.43E-02 |
| Coagulation Factor V | F5 | P12259 | -0.032 | 1.49E-02 |
| Tyrosine-protein kinase receptor Tie-1; soluble | TIE1 | P35590 | -0.054 | 1.63E-02 |
| Mitogen-activated protein kinase 14 | MAPK14 | Q16539 | -0.066 | 1.67E-02 |
| Tyrosine-protein kinase Yes | YES1 | P07947 | -0.053 | 1.67E-02 |
| Protein kinase C theta type | PRKCQ | Q04759 | -0.092 | 1.75E-02 |
| Proteasome subunit alpha type-2 | PSMA2 | P25787 | 0.062 | 1.75E-02 |
| N-acylethanolamine-hydrolyzing acid amidase | NAAA | Q02083 | 0.069 | 1.76E-02 |
| Trefoil factor 3 | TFF3 | Q07654 | 0.042 | 1.77E-02 |
| N-acetyl-D-glucosamine kinase | NAGK | Q9UJ70 | -0.074 | 1.81E-02 |

| Protein | Entrez Gene Symbol | UniProt ID | log2-Fold Change | FDR p-value |
|---|---|---|---|---|
| Cathepsin Z | CTSZ | Q9UBR2 | 0.058 | 1.91E-02 |
| Ficolin-2 | FCN2 | Q15485 | -0.043 | 1.97E-02 |
| Ephrin-A4 | EFNA4 | P52798 | 0.060 | 1.97E-02 |
| Interleukin-17 receptor B | IL17RB | Q9NRM6 | 0.063 | 1.97E-02 |
| Platelet glycoprotein VI | GP6 | Q9HCN6 | -0.061 | 1.97E-02 |
| Calcium-dependent phospholipase A2 | PLA2G5 | P39877 | -0.039 | 2.07E-02 |
| C-C motif chemokine 15 | CCL15 | Q16663 | 0.043 | 2.09E-02 |
| cGMP-specific 3';5'-cyclic phosphodiesterase | PDE5A | O76074 | -0.155 | 2.09E-02 |
| Calcium/calmodulin-dependent protein kinase kinase 1 | CAMKK1 | Q8N5S9 | -0.097 | 2.11E-02 |
| Kremen protein 2 | KREMEN2 | Q8NCW0 | -0.068 | 2.16E-02 |
| Cytokine receptor-like factor 2 | CRLF2 | Q9HC73 | -0.054 | 2.19E-02 |
| ATP synthase subunit beta; mitochondrial | ATP5B | P06576 | -0.054 | 2.21E-02 |
| Kunitz-type protease inhibitor 2 | SPINT2 | O43291 | -0.052 | 2.32E-02 |
| Creatine kinase M-type:Creatine kinase B-type heterodimer | CKB CKM | P12277 P06732 | -0.121 | 2.35E-02 |
| AH receptor-interacting protein | AIP | O00170 | -0.113 | 2.35E-02 |
| Calpain I | CAPN1 CAPNS1 | P07384 P04632 | -0.096 | 2.35E-02 |
| B-cell receptor CD22 | CD22 | P20273 | -0.030 | 2.57E-02 |
| Afamin | AFM | P43652 | -0.021 | 2.57E-02 |
| Apolipoprotein D | APOD | P05090 | -0.042 | 2.57E-02 |
| A disintegrin and metalloproteinase with thrombospondin motifs 15 | ADAMTS15 | Q8TE58 | -0.039 | 2.60E-02 |
| Junctional adhesion molecule-like | AMICA1 | Q86YT9 | 0.021 | 2.60E-02 |
| Macrophage-capping protein | CAPG | P40121 | 0.088 | 2.60E-02 |
| Leukotriene A-4 hydrolase | LTA4H | P09960 | -0.313 | 2.60E-02 |
| Stress-induced-phosphoprotein 1 | STIP1 | P31948 | -0.117 | 2.74E-02 |
| Glyceraldehyde-3-phosphate dehydrogenase | GAPDH | P04406 | -0.145 | 2.76E-02 |
| Carbohydrate sulfotransferase 2 | CHST2 | Q9Y4C5 | -0.041 | 2.76E-02 |
| C-C motif chemokine 17 | CCL17 | Q92583 | -0.116 | 2.81E-02 |
| Calcium/calmodulin-dependent protein kinase type 1D | CAMK1D | Q8IU85 | -0.055 | 2.98E-02 |
| Lipopolysaccharide-binding protein | LBP | P18428 | 0.073 | 2.98E-02 |
| Leukocyte immunoglobulin-like receptor subfamily B member 1 | LILRB1 | Q8NHL6 | 0.032 | 2.98E-02 |
| Insulin-like growth factor-binding protein 1 | IGFBP1 | P08833 | 0.123 | 3.23E-02 |
| Brain natriuretic peptide 32 | NPPB | P16860 | 0.048 | 3.28E-02 |
| Eukaryotic translation initiation factor 5 | EIF5 | P55010 | 0.078 | 3.34E-02 |

| Protein | Entrez Gene Symbol | UniProt ID | log2-Fold Change | FDR p-value |
|---|---|---|---|---|
| Plasma kallikrein | KLKB1 | P03952 | -0.041 | 3.36E-02 |
| Dual specificity protein phosphatase 3 | DUSP3 | P51452 | -0.087 | 3.36E-02 |
| Coagulation factor Xa | F10 | P00742 | 0.029 | 3.41E-02 |
| Peptide YY | PYY | P10082 | 0.088 | 3.42E-02 |
| Low affinity immunoglobulin epsilon Fc receptor | FCER2 | P06734 | 0.049 | 3.42E-02 |
| Tyrosine-protein kinase Tec | TEC | P42680 | -0.088 | 3.47E-02 |
| Complement C4 | C4A C4B | P0C0L4; P0C0L5 | -0.038 | 3.48E-02 |
| Caspase-10 | CASP10 | Q92851 | -0.036 | 3.62E-02 |
| Matrilin-2 | MATN2 | O00339 | 0.031 | 3.62E-02 |
| Fibroblast growth factor 6 | FGF6 | P10767 | -0.073 | 3.62E-02 |
| 72 kDa type IV collagenase | MMP2 | P08253 | 0.036 | 3.83E-02 |
| Serum paraoxonase/arylesterase 1 | PON1 | P27169 | -0.035 | 3.84E-02 |
| Ectonucleoside triphosphate diphosphohydrolase 5 | ENTPD5 | O75356 | -0.022 | 3.85E-02 |
| Glycylpeptide N-tetradecanoyltransferase 1 | NMT1 | P30419 | -0.084 | 3.94E-02 |
| Cadherin-3 | CDH3 | P22223 | -0.029 | 3.94E-02 |
| Histone-lysine N-methyltransferase EHMT2 | EHMT2 | Q96KQ7 | -0.060 | 3.98E-02 |
| Hsp90 co-chaperone Cdc37 | CDC37 | Q16543 | -0.064 | 4.04E-02 |
| Intercellular adhesion molecule 5 | ICAM5 | Q9UMF0 | 0.029 | 4.05E-02 |
| Vacuolar protein sorting-associated protein VTA1 homolog | VTA1 | Q9NP79 | -0.118 | 4.06E-02 |
| Sialic acid-binding Ig-like lectin 7 | SIGLEC7 | Q9Y286 | 0.032 | 4.06E-02 |
| Bcl-2-like protein 1 | BCL2L1 | Q07817 | -0.041 | 4.07E-02 |
| Cofilin-1 | CFL1 | P23528 | -0.106 | 4.20E-02 |
| Osteomodulin | OMD | Q99983 | 0.065 | 4.26E-02 |
| Heterogeneous nuclear ribonucleoprotein Q | SYNCRIP | O60506 | -0.053 | 4.47E-02 |
| E3 ubiquitin-protein ligase Mdm2 | MDM2 | Q00987 | -0.045 | 4.47E-02 |
| Low affinity immunoglobulin gamma Fc region receptor II-b | FCGR2B | P31994 | 0.044 | 4.47E-02 |
| Retinol-binding protein 4 | RBP4 | P02753 | -0.039 | 4.52E-02 |
| Transforming growth factor beta-1 | TGFB1 | P01137 | -0.039 | 4.53E-02 |
| Histone deacetylase 8 | HDAC8 | Q9BY41 | -0.027 | 4.53E-02 |
| Tyrosine-protein kinase transmembrane receptor ROR1 | ROR1 | Q01973 | 0.022 | 4.61E-02 |
| Cystatin-C | CST3 | P01034 | 0.019 | 4.61E-02 |
| Creatine kinase B-type | CKB | P12277 | 0.029 | 4.61E-02 |
| GTP-binding nuclear protein Ran | RAN | P62826 | -0.184 | 4.81E-02 |

| Protein | Entrez Gene Symbol | UniProt ID | log2-Fold Change | FDR p-value |
|---|---|---|---|---|
| Tumor necrosis factor-inducible gene 6 protein | TNFAIP6 | P98066 | 0.040 | 4.81E-02 |
| Desmoglein-1 | DSG1 | Q02413 | -0.026 | 4.95E-02 |

**Supplemental Table III: Top 10 pathways among the 200 proteins significantly altered by torcetrapib identified by Reactome Pathway Database.**

| Pathway Name | # Entities Found | # Entities Total | Entities FDR |
|---|---|---|---|
| Signaling by Interleukins† | 43 | 706 | 4.13E-09 |
| Immune System† | 80 | 2444 | 9.17E-06 |
| Cytokine Signaling in Immune System† | 45 | 1013 | 9.17E-06 |
| Platelet activation, signaling and aggregation | 21 | 279 | 1.95E-05 |
| Toll Like Receptor TLR6:TLR2 Cascade† | 12 | 95 | 3.91E-05 |
| MyD88:Mal cascade initiated on plasma membrane† | 12 | 95 | 3.91E-05 |
| Toll Like Receptor 2 (TLR2) Cascade† | 12 | 98 | 4.34E-05 |
| Toll Like Receptor TLR1:TLR2 Cascade† | 12 | 98 | 4.34E-05 |
| Regulation of Insulin-like Growth Factor (IGF) transport and uptake | 13 | 124 | 7.21E-05 |
| Innate Immune System† | 53 | 1489 | 1.12E-04 |

There is significant enrichment of pathways involving inflammatory and immune functions. Number of entities found is the number of proteins and interacting molecules common between the submitted list of significant proteins and the pathway. Number of entities total is the number of proteins and interactions within a given pathway. Entities FDR is the false discovery rate corrected over-representation probability. †Indicates pathways involved in inflammation or immunity.

**Supplemental Table IV: Top 20 KEGG and BIOCARTA Pathways of the 200 proteins significantly altered by torcetrapib identified by DAVID.**

| Pathway Database | Pathway | Count | p-value |
|---|---|---|---|
| KEGG | B cell receptor signaling pathway† | 10 | 8.7E-04 |
| KEGG | Fc gamma R-mediated phagocytosis† | 11 | 1.8E-03 |
| BioCarta | BCR signaling pathway† | 8 | 2.5E-03 |
| BioCarta | Fc epsilon receptor I signaling in mast cells† | 8 | 4.9E-03 |
| BioCarta | Phospholipids as signaling intermediaries† | 8 | 4.9E-03 |
| BioCarta | fMLP induced chemokine gene expression in HMC-1 cells† | 6 | 5.8E-03 |
| BioCarta | Regulation of eIF4e and p70 S6 Kinase | 7 | 7.9E-03 |
| KEGG | VEGF signaling pathway | 12 | 8.8E-03 |
| KEGG | Adipocytokine signaling pathway | 8 | 1.3E-02 |
| BioCarta | Ras signaling pathway | 5 | 2.0E-02 |
| KEGG | Viral Carcinogenesis | 10 | 2.3E-02 |
| BioCarta | Transcription factor CREB and its extracellular signals | 7 | 2.4E-02 |
| KEGG | cGMP-PKG signaling pathway | 7 | 3.3E-02 |
| BioCarta | NFAT and hypertrophy of the heart | 8 | 3.3E-02 |
| KEGG | Platelet activation† | 9 | 3.6E-02 |
| KEGG | Acute myeloid leukemia† | 6 | 3.6E-2 |
| KEGG | Shigellosis† | 6 | 3.6E-02 |
| BioCarta | Influence of Ras and Rho proteins on G1 to S transition | 6 | 4.0E-02 |

| | | | |
|---|---|---|---|
| KEGG | Alzheimer's disease | 8 | 4.2E-02 |
| KEGG | Non-alcoholic fatty liver disease | 9 | 4.8E-02 |

There is significant enrichment in pathways involving inflammatory and immune system functions, corroborating our findings from Ingenuity Pathway Analysis. Count is the number of significant proteins found in a given pathway. P-value is by DAVID using a modified Fisher Exact p-value. †Indicates pathways involved in inflammation or immunity.

**Supplemental Table V: Proteins that changed significantly in a univariate analysis at 3 months compared to baseline for the atorvastatin only treatment arm**, truncated at a false discovery rate adjusted p-value <0.05.

| Protein | EntrezGene Symbol | UniProt ID | log2-Fold Change | FDR p-value |
|---|---|---|---|---|
| Hepcidin | HAMP | P81172 | -0.245 | 2.92E-03 |
| Stromal cell-derived factor 1 | CXCL12 | P48061 | -0.069 | 7.84E-03 |
| C-C motif chemokine 15 | CCL15 | Q16663 | 0.063 | 7.84E-03 |
| Leptin receptor | LEPR | P48357 | -0.061 | 1.02E-02 |
| Kunitz-type protease inhibitor 2 | SPINT2 | O43291 | -0.089 | 1.61E-02 |
| Beta-2-microglobulin | B2M | P61769 | -0.033 | 1.61E-02 |
| Complement C1r subcomponent | C1R | P00736 | -0.108 | 2.20E-02 |
| Mitogen-activated protein kinase 1 | MAPK1 | P28482 | -0.227 | 2.37E-02 |
| Tumor necrosis factor ligand superfamily member 12 | TNFSF12 | O43508 | -0.062 | 3.62E-02 |
| E-selectin | SELE | P16581 | 0.054 | 3.62E-02 |
| Methionine aminopeptidase 2 | METAP2 | P50579 | -0.196 | 3.82E-02 |
| Ribosome maturation protein SBDS | SBDS | Q9Y3A5 | -0.225 | 3.82E-02 |
| AMP Kinase (alpha2beta2gamma1) | PRKAA2 PRKAB2 PRKAG1 | P54646 O43741 P54619 | -0.126 | 3.82E-02 |
| Calcium/calmodulin-dependent protein kinase type II subunit delta | CAMK2D | Q13557 | -0.221 | 4.84E-02 |
| N-acetyl-D-glucosamine kinase | NAGK | Q9UJ70 | -0.069 | 4.95E-02 |
| Peptidyl-prolyl cis-trans isomerase D | PPID | Q08752 | -0.380 | 4.95E-02 |
| Heterogeneous nuclear ribonucleoprotein A/B | HNRNPAB | Q99729 | -0.106 | 4.95E-02 |
| Tyrosine-protein kinase Tec | TEC | P42680 | -0.156 | 4.95E-02 |

**Supplemental Table VI: The top Ingenuity Pathway Analysis (IPA) 'Canonical Pathways' of the proteins significantly altered by torcetrapib plus atorvastatin with removal of the proteins significantly altered by atorvastatin only.**

| IPA Canonical Pathways | p-value | Ratio* |
|---|---|---|
| B Cell Receptor Signaling† | 0.035 | 0.370 (17/46) |
| Calcium Signaling | 0.015 | 0.467 (7/15) |
| RAR Activation† | 0.020 | 0.367 (11/30) |
| Role of NFAT in Regulation of the Immune Response† | 0.024 | 0.370 (10/27) |
| PPARα/RXRα Activation | 0.026 | 0.343 (12/35) |
| PI3K Signaling in B Lymphocytes† | 0.031 | 0.357 (10/28) |
| FXR/RXR Activation | 0.032 | 0.334 (11/32) |
| Sphingosine-1-phosphate Signaling† | 0.036 | 0.381 (8/21) |
| PI3K/AKT Signaling | 0.040 | 0.381 (8/21) |
| CXCR4 Signaling† | 0.040 | 0.345 (10/29) |
| TREM1 Signaling† | 0.044 | 0.389 (7/18) |

*The ratio indicates the number of significant proteins that map to the canonical pathway divided by the total number of proteins measured in our study that map to the same pathway. †Indicates pathways identified by IPA as involved in inflammation or immunity.

**Supplemental Table VII: Proteins previously shown to be prognostic of cardiovascular events[1] that changed between baseline and 3 months.**

| Protein | Entrez Gene Symbol | UniProt ID | log2-Fold Change | FDR p-value | HR per SD[1] |
|---|---|---|---|---|---|
| **TORCETRAPIB TREATMENT** | | | | | |
| **Prognostic proteins moving in the direction of increased risk** | | | | | |
| Neurogenic locus notch homolog protein 3 | NOTCH3 | Q9UM47 | 0.103 | 3.15E-06 | 1.21 |
| Thrombospondin-2 | THBS2 | P35442 | 0.143 | 1.95E-05 | 1.54 |
| **Troponin I, cardiac muscle** | TNNI3 | P19429 | 0.118 | 3.01E-05 | 1.27 |
| Dual 3',5'-cyclic-AMP and -GMP phosphodiesterase 11A | PDE11A | Q9HCR9 | -0.123 | 2.79E-04 | 0.69 |
| Follistatin-related protein 3 | FSTL3 | O95633 | 0.058 | 2.79E-04 | 1.56 |
| **Alpha-2-antiplasmin** | SERPINF2 | P08697 | -0.047 | 9.27E-04 | 0.64 |
| Spondin-1 | SPON1 | Q9HCB6 | 0.063 | 1.24E-03 | 1.42 |
| Beta-Ala-His dipeptidase | CNDP1 | Q96KN2 | -0.082 | 1.96E-03 | 0.69 |
| **Angiopoietin-2** | ANGPT2 | O15123 | 0.068 | 2.30E-03 | 1.67 |
| Interleukin-8 | CXCL8 | P10145 | 0.057 | 2.95E-03 | 1.19 |
| Mannose-binding protein C | MBL2 | P11226 | 0.067 | 3.93E-03 | 1.24 |
| Heat shock 70 kDa protein 1A/1B | HSPA1A | P08107 | 0.08 | 4.40E-03 | 1.34 |
| Interleukin-1 receptor-like 1 | IL1RL1 | Q01638 | 0.06 | 6.11E-03 | 1.31 |
| Hepatitis A virus cellular receptor 2 | HAVCR2 | Q8TDQ0 | 0.057 | 6.89E-03 | 1.61 |
| Growth hormone receptor | GHR | P10912 | -0.05 | 7.30E-03 | 0.63 |
| Cytochrome P450 3A4 | CYP3A4 | P08684 | -0.107 | 7.55E-03 | 0.79 |
| Insulin-like growth factor-binding protein 2 | IGFBP2 | P18065 | 0.099 | 8.40E-03 | 1.58 |
| Tumor necrosis factor ligand superfamily member 12 | TNFSF12 | O43508 | -0.035 | 1.10E-02 | 0.74 |
| 6-phosphogluconate dehydrogenase, decarboxylating | PGD | P52209 | -0.307 | 1.30E-02 | 0.81 |

| Protein | Entrez Gene Symbol | UniProt ID | log2-Fold Change | FDR p-value | HR per SD[1] |
|---|---|---|---|---|---|
| Trefoil factor 3 | TFF3 | Q07654 | 0.042 | 1.77E-02 | 1.3 |
| Cathepsin Z | CTSZ | Q9UBR2 | 0.058 | 1.91E-02 | 1.32 |
| Ficolin-2 | FCN2 | Q15485 | -0.043 | 1.97E-02 | 0.83 |
| Ephrin-A4 | EFNA4 | P52798 | 0.06 | 1.97E-02 | 1.44 |
| Creatine kinase M-type:Creatine kinase B-type heterodimer | CKB CKM | P12277 P06732 | -0.121 | 2.35E-02 | 0.69 |
| Afamin | AFM | P43652 | -0.021 | 2.57E-02 | 0.81 |
| Macrophage-capping protein | CAPG | P40121 | 0.088 | 2.60E-02 | 1.36 |
| Lipopolysaccharide-binding protein | LBP | P18428 | 0.073 | 2.98E-02 | 1.27 |
| Insulin-like growth factor-binding protein 1 | IGFBP1 | P08833 | 0.123 | 3.23E-02 | 1.29 |
| Brain natriuretic peptide 32 | NPPB | P16860 | 0.048 | 3.28E-02 | 1.21 |
| Plasma kallikrein | KLKB1 | P03952 | -0.041 | 3.36E-02 | 0.76 |
| Matrilin-2 | MATN2 | O00339 | 0.031 | 3.62E-02 | 1.26 |
| Ectonucleoside triphosphate diphosphohydrolase 5 | ENTPD5 | O75356 | -0.022 | 3.85E-02 | 0.8 |
| Cadherin-3 | CDH3 | P22223 | -0.029 | 3.94E-02 | 0.67 |
| Intercellular adhesion molecule 5 | ICAM5 | Q9UMF0 | 0.029 | 4.05E-02 | 1.21 |
| Low affinity immunoglobulin gamma Fc region receptor II-a/b | FCGR2B | P12318 P31994 | 0.044 | 4.47E-02 | 1.24 |
| Tyrosine-protein kinase transmembrane receptor ROR1 | ROR1 | Q01973 | 0.022 | 4.61E-02 | 1.24 |
| Cystatin-C | CST3 | P01034 | 0.019 | 4.61E-02 | 1.49 |
| **Prognostic proteins moving in the direction of reduced risk** | | | | | |
| Carbonic anhydrase 6 | CA6 | P23280 | 0.304 | 8.88E-23 | 0.82 |
| Renin | REN | P00797 | -0.316 | 8.65E-13 | 1.29 |

| Protein | Entrez Gene Symbol | UniProt ID | log2-Fold Change | FDR p-value | HR per SD[1] |
|---|---|---|---|---|---|
| 14-3-3 protein family | YWHAB,YWHAE,YWHAG,YWHAH,YWHAQ,YWHAZ,SFN | P31946, P62258, P61981, Q04917, P27348, P63104, P31947 | -0.168 | 3.15E-03 | 1.19 |
| Parathyroid hormone-related protein | PTHLH | P12272 | -0.055 | 7.01E-03 | 1.17 |
| Pappalysin-1 | PAPPA | Q13219 | -0.048 | 1.16E-02 | 1.29 |
| Insulin-like growth factor-binding protein 6 | IGFBP6 | P24592 | -0.042 | 1.23E-02 | 1.26 |
| **C-C motif chemokine 18** | CCL18 | P55774 | -0.069 | 1.36E-02 | 1.47 |
| Tyrosine-protein kinase Yes | YES1 | P07947 | -0.053 | 1.67E-02 | 1.2 |
| N-acetyl-D-glucosamine kinase | NAGK | Q9UJ70 | -0.074 | 1.81E-02 | 1.23 |
| Platelet glycoprotein VI | GP6 | Q9HCN6 | -0.061 | 1.97E-02 | 1.32 |
| Kunitz-type protease inhibitor 2 | SPINT2 | O43291 | -0.052 | 2.32E-02 | 1.17 |
| Coagulation factor Xa | F10 | P00742 | 0.029 | 3.41E-02 | 0.8 |

| | | | | | |
|---|---|---|---|---|---|
| **ATORVASTATIN TREATMENT** | | | | | |
| **Prognostic proteins moving in the direction of reduced risk** | | | | | |
| Stromal cell-derived factor 1 | CXCL12 | P48061 | -0.069 | 7.84E-03 | 1.28 |
| Kunitz-type protease inhibitor 2 | SPINT2 | O43291 | -0.089 | 1.61E-02 | 1.17 |
| Beta-2-microglobulin | B2M | P61769 | -0.033 | 1.61E-02 | 1.54 |
| N-acetyl-D-glucosamine kinase | NAGK | Q9UJ70 | -0.069 | 4.95E-02 | 1.23 |
| **Prognostic proteins moving in the direction of increased risk** | | | | | |
| Tumor necrosis factor ligand superfamily member 12 | TNFSF12 | O43508 | -0.062 | 3.62E-02 | 0.74 |

Subset of 49 (torcetrapib) and 5 (atorvastatin) of the 200 proteins previously found to be prognostic of cardiovascular events[1] which changed within the treatment arm at 3 months compared to baseline, in rank order of p-values. False discovery rate (FDR) correction was applied to the p-values for 986 protein measurements and FDR p-values <0.05 are reported. The 4 proteins listed in bold font are included in the 9-protein risk model. The univariate hazard ratios in the last column are hazard ratios (HR) per standard deviation (SD) of their association with cardiovascular and mortality outcomes in patients with stable coronary heart disease, previously published.[1] Protein lists are divided into those that move in the direction of increased or decreased risk by treatment arm in the current study compared to the published reference.[1]

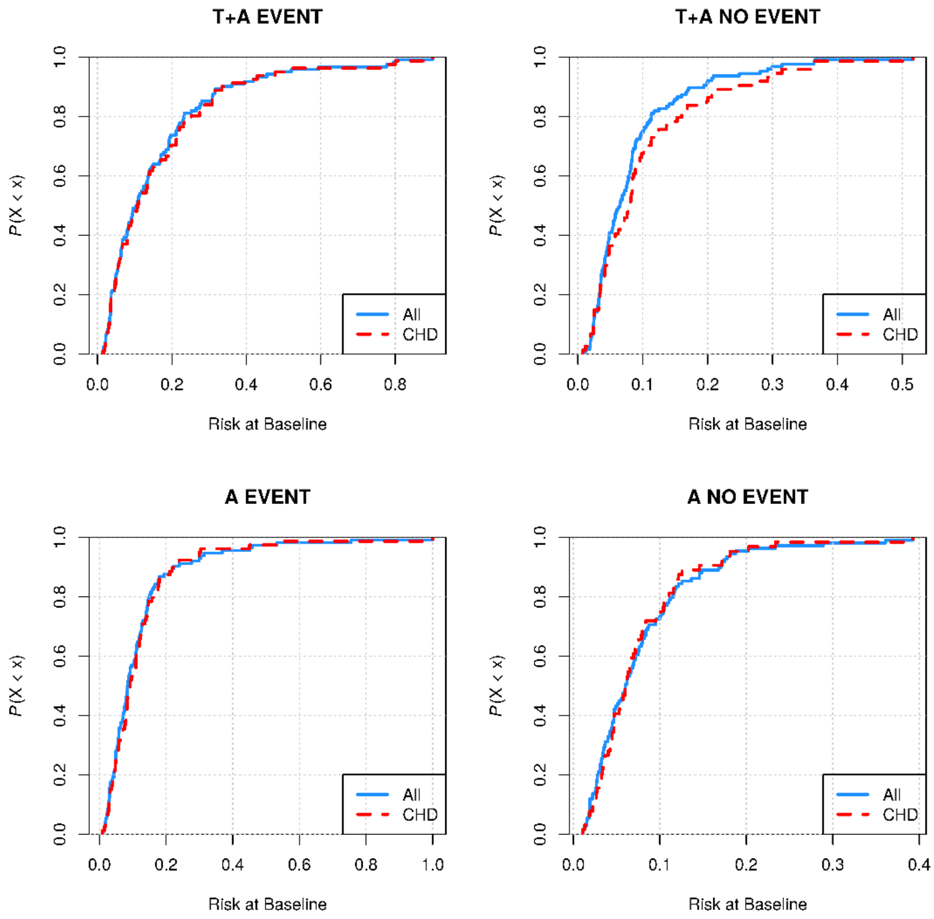


**Supplemental Figure I: Comparison of risk score distributions in participants with known coronary heart disease (CHD; red) with risk score distribution in all participants (including diabetic participants enrolled in the study without known CHD) (blue).** T = torcetrapib; A = atorvastatin.

## B Cell Receptor Signaling

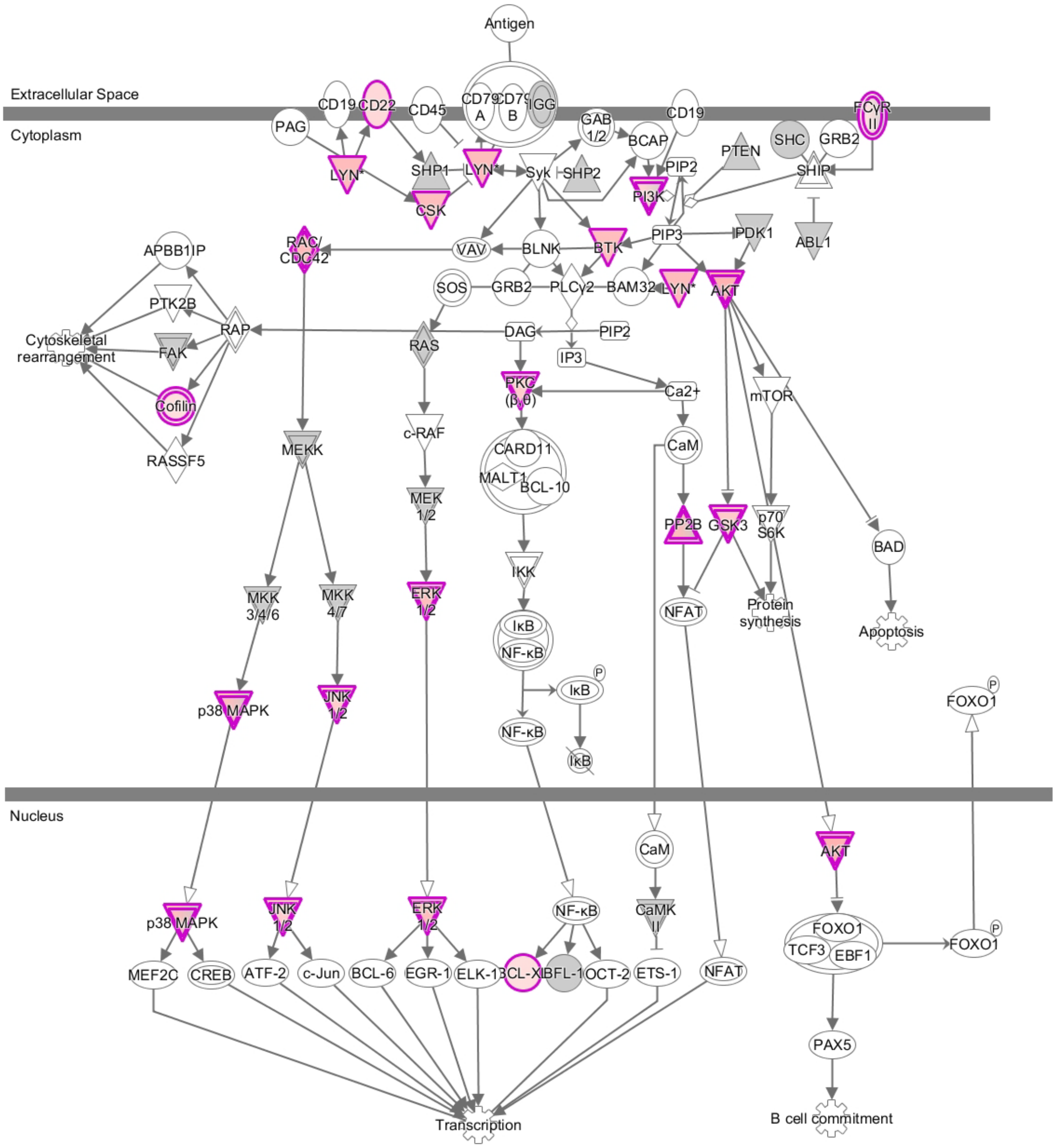


**Supplemental Figure II: B cell receptor signaling canonical pathway from Ingenuity Pathway Analysis.** Nodes represent gene symbol name, corresponding to protein measured. The intensity of the node color, ranging from light purple to dark red, indicates degree of significance of FDR p-value, with dark red indicating a higher degree of significance. White nodes represent genes in the IPA canonical pathway that were not significant or not measured. The node shapes denote complex/group ( ),

chemical (), cytokine (), disease (), enzyme (), G-protein coupled receptor (), growth factor (), ion channel (), kinase (), ligand-dependent nuclear receptor (), peptidase (), phosphatase (), transcription regulator (), transmembrane receptor (), transporter (), and other ().

## Calcium Signaling

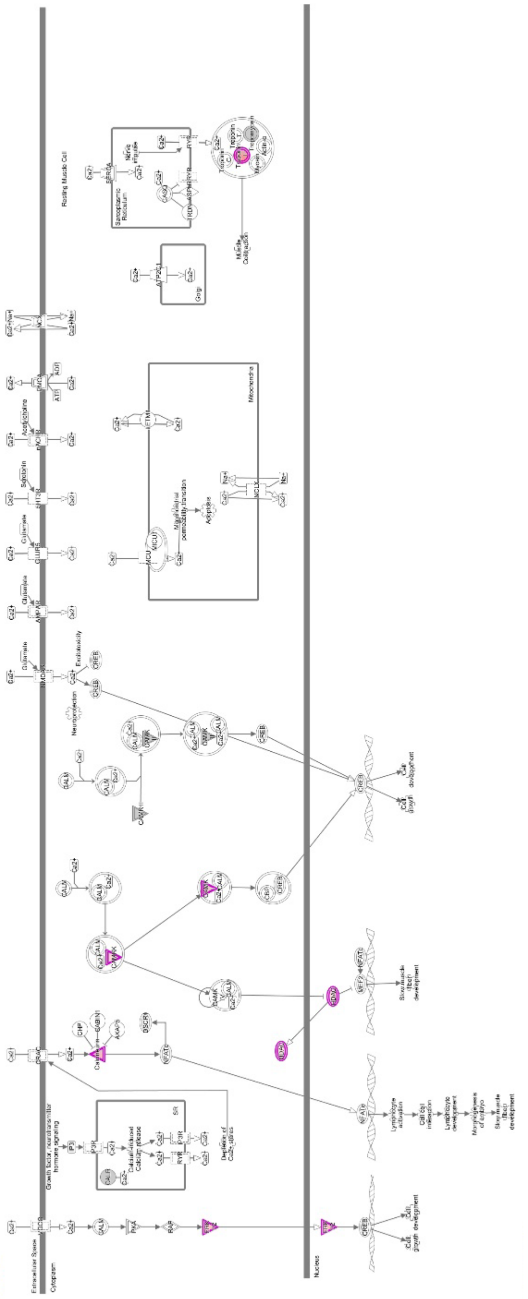

**Supplemental Figure III: Calcium signaling canonical pathway from Ingenuity Pathway Analysis.** Nodes represent gene symbol name, corresponding to protein measured. The intensity of the node color, ranging from light purple to dark red, indicates degree of significance of FDR p-value, with dark red indicating a higher degree of significance. White nodes represent genes in the IPA canonical pathway

that were not significant or not measured. The node shapes denote complex/group ( ), chemical ( ), cytokine ( ), disease ( ), enzyme ( ), G-protein coupled receptor ( ), growth factor ( ), ion channel ( ), kinase ( ), ligand-dependent nuclear receptor ( ), peptidase ( ), phosphatase ( ), transcription regulator ( ), transmembrane receptor ( ), transporter ( ), and other ( ).

## PPARα/RXRα Activation

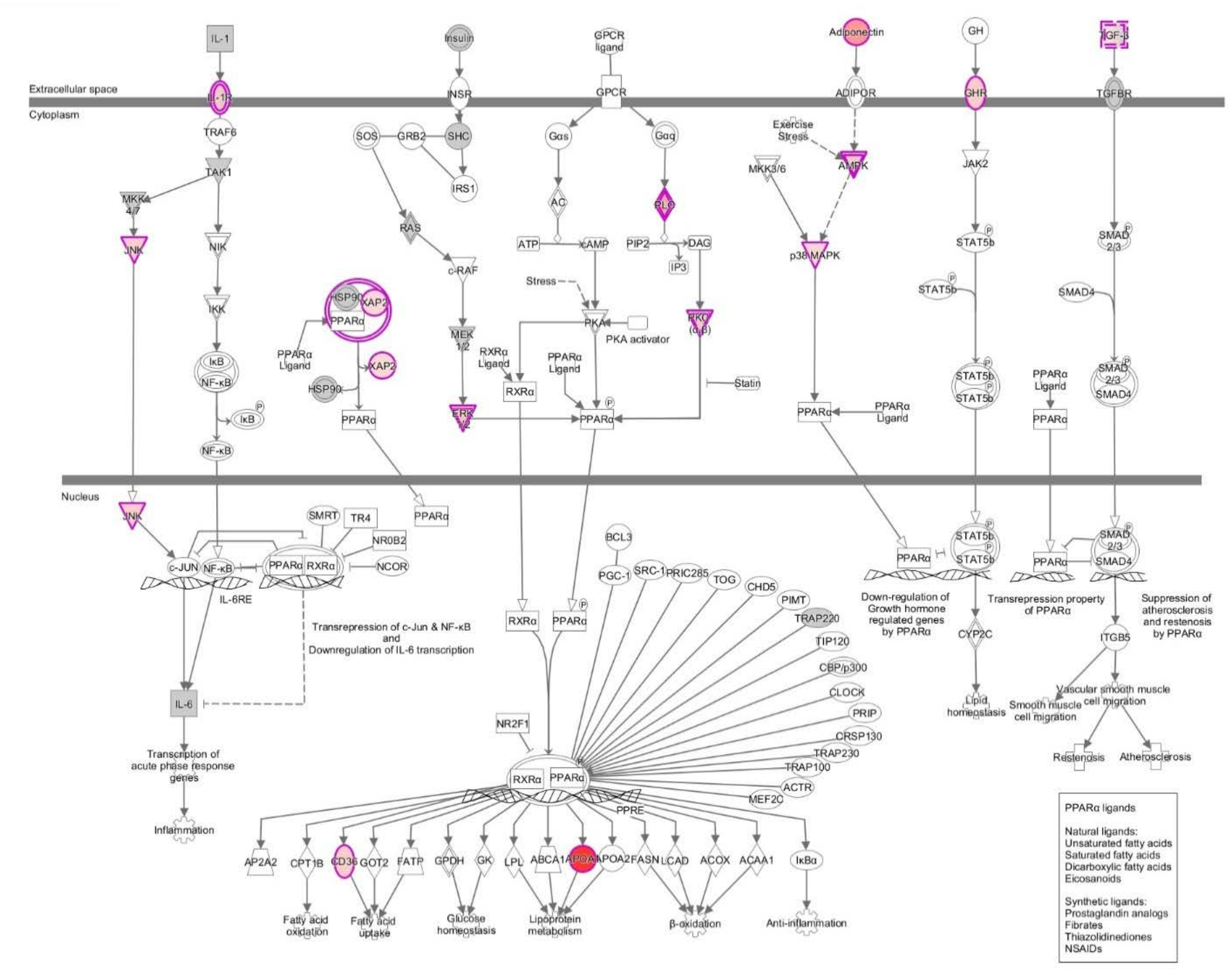


**Supplemental Figure IV: PPARα/RXRα activation canonical pathway from Ingenuity Pathway Analysis.** Nodes represent gene symbol name, corresponding to protein measured. The intensity of the node color, ranging from light purple to dark red, indicates degree of significance of FDR p-value, with dark red indicating a higher degree of significance. White nodes represent genes in the IPA canonical pathway that were not significant or not measured. The node shapes denote complex/group (◎), chemical (⬭), cytokine (□), disease (✚), enzyme (◇), G-protein coupled receptor (▯), growth factor (⬚), ion channel (▯), kinase (▽), ligand-dependent nuclear receptor (▭), peptidase (◇), phosphatase (△), transcription regulator (⬭), transmembrane receptor (0), transporter (⏢), and other (○).

## RAR Activation

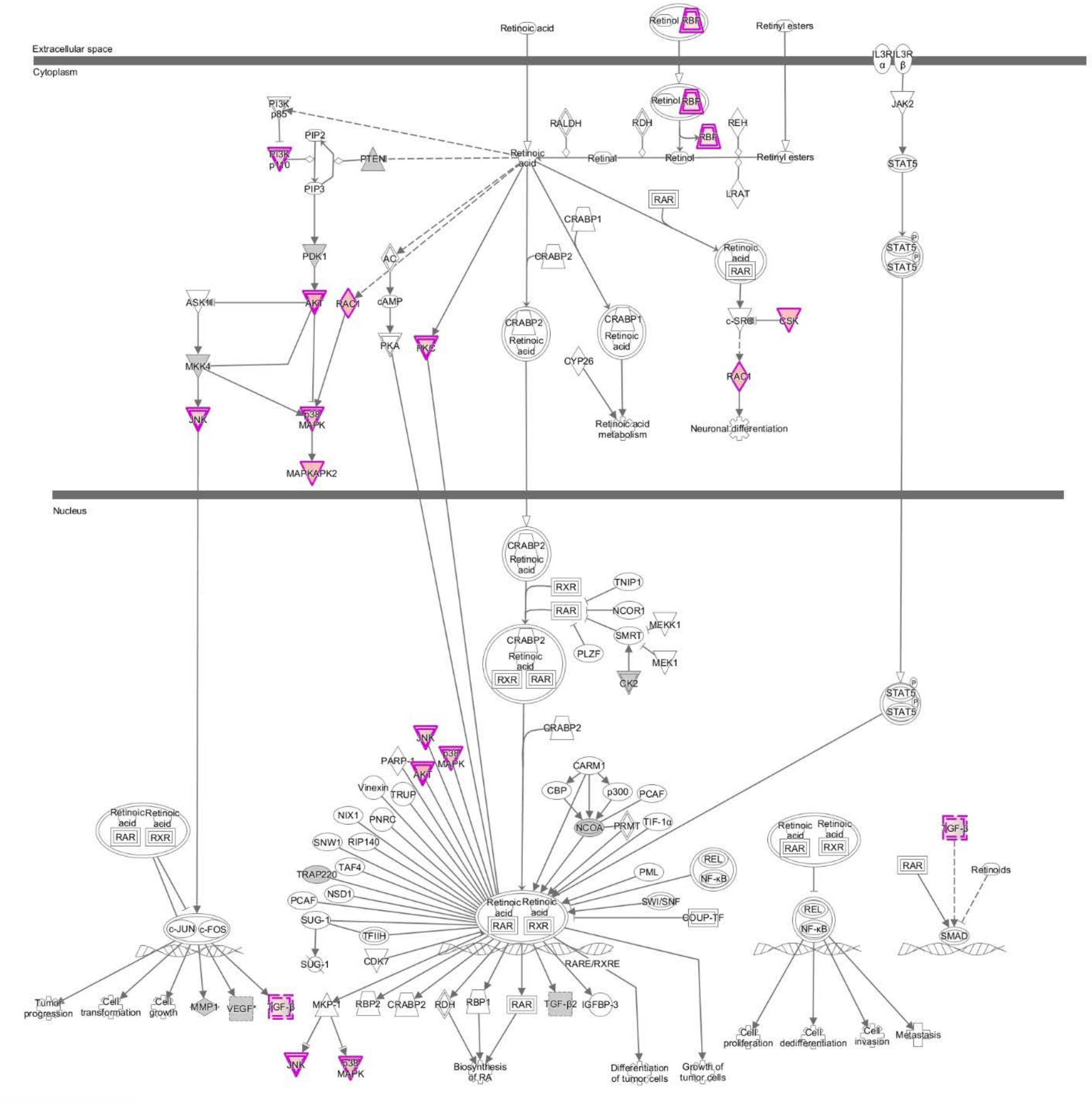


**Supplemental Figure V: RAR activation canonical pathway from Ingenuity Pathway Analysis.** Nodes represent gene symbol name, corresponding to protein measured. The intensity of the node color, ranging from light purple to dark red, indicates degree of significance of FDR p-value, with dark red indicating a higher degree of significance. White nodes represent genes in the IPA canonical pathway that were not significant or not measured. The node shapes denote complex/group ( ), chemical (

), cytokine ( ), disease ( ), enzyme ( ), G-protein coupled receptor ( ), growth factor ( ), ion channel ( ), kinase ( ), ligand-dependent nuclear receptor ( ), peptidase ( ), phosphatase ( ), transcription regulator ( ), transmembrane receptor ( ), transporter ( ), and other ( ).

**Role of NFAT in Regulation of the Immune Response**

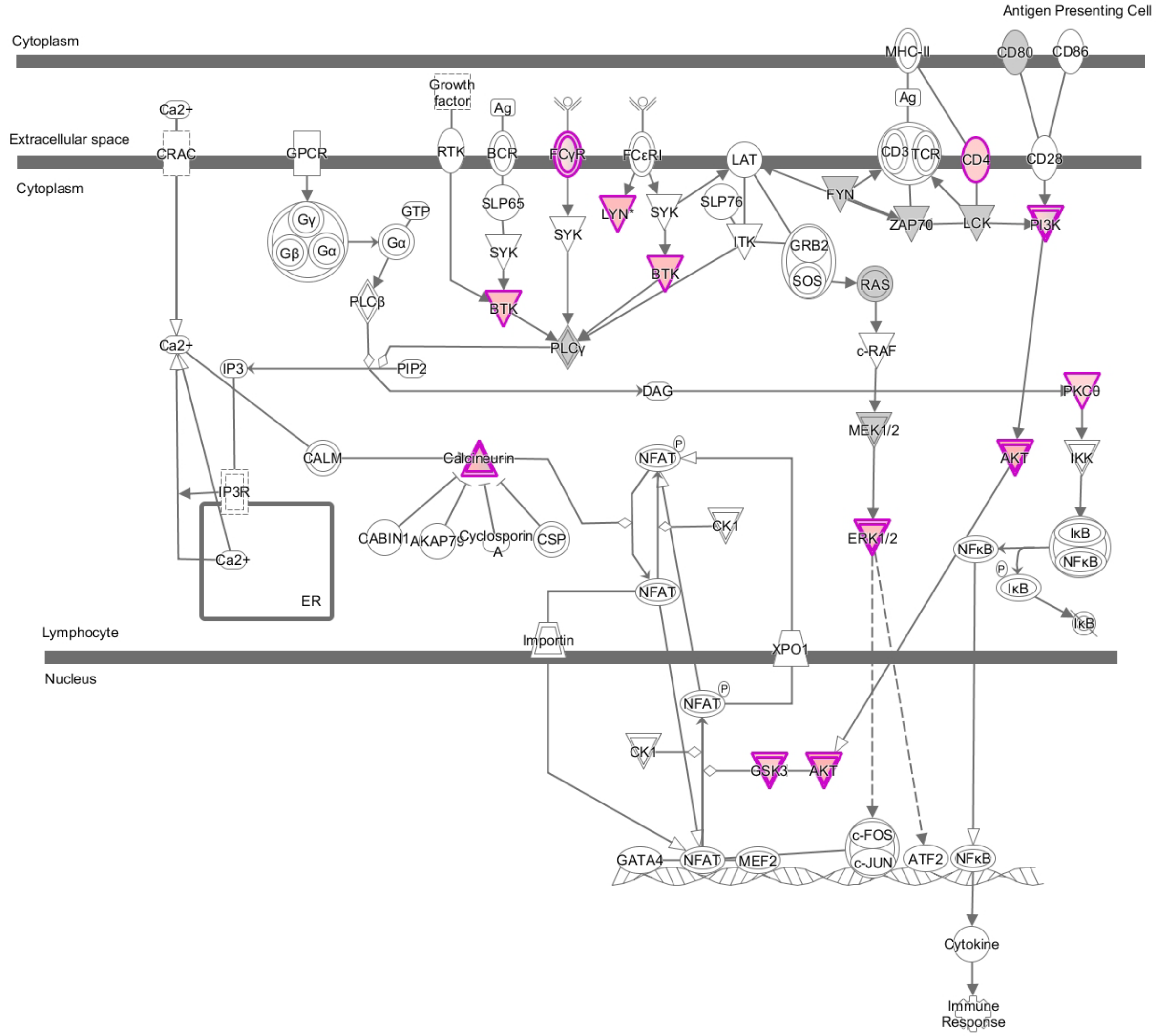


**Supplemental Figure VI: Role of NFAT in regulation of the immune response canonical pathway from Ingenuity Pathway Analysis.** Nodes represent gene symbol name, corresponding to protein measured. The intensity of the node color, ranging from light purple to dark red, indicates degree of significance of FDR p-value, with dark red indicating a higher degree of significance. White nodes represent genes in the IPA canonical pathway that were not significant or not measured. The node shapes denote complex/group (◎), chemical (⬭), cytokine (□), disease (✚), enzyme (◇), G-protein coupled receptor (▯), growth factor (⬚), ion channel (▯), kinase (▽), ligand-dependent nuclear receptor (▭),

peptidase (◇), phosphatase (△), transcription regulator (⬭), transmembrane receptor (⬯), transporter (⏢), and other (○).

## PI3K Signaling in B Lymphocytes

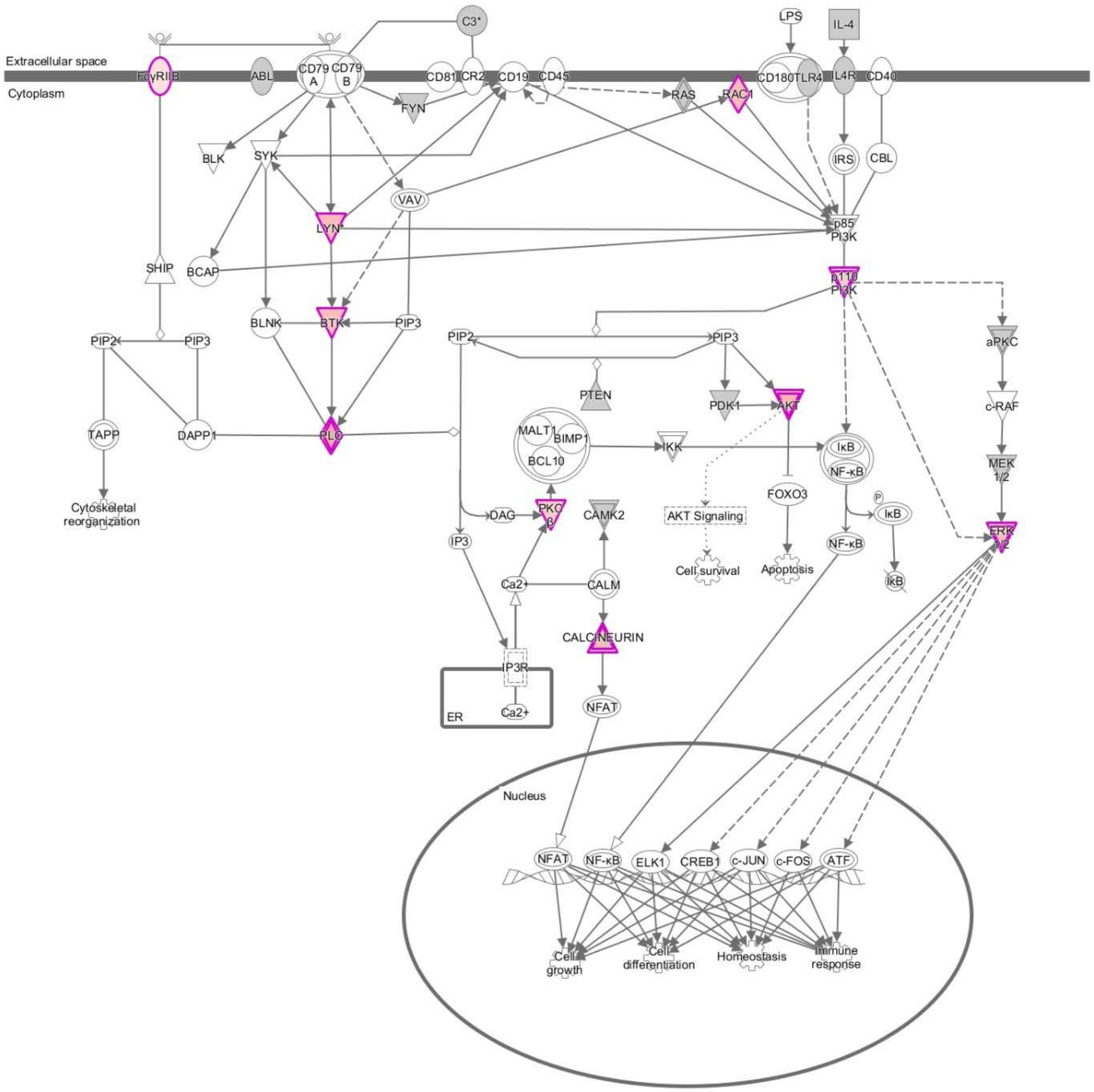


**Supplemental Figure VII: PI3K signaling in B lymphocytes canonical pathway from Ingenuity Pathway Analysis.** Nodes represent gene symbol name, corresponding to protein measured. The intensity of the node color, ranging from light purple to dark red, indicates degree of significance of FDR p-value, with dark red indicating a higher degree of significance. White nodes represent genes in the IPA canonical pathway that were not significant or not measured. The node shapes denote complex/group (◎), chemical (⬭), cytokine (□), disease (✚), enzyme (◇), G-protein coupled receptor (▯), growth factor

(⬚), ion channel (⬚), kinase (▽), ligand-dependent nuclear receptor (▭), peptidase (◇), phosphatase (△), transcription regulator (⬭), transmembrane receptor (⬯), transporter (⏢), and other (○).

## T-Cell Receptor Signaling

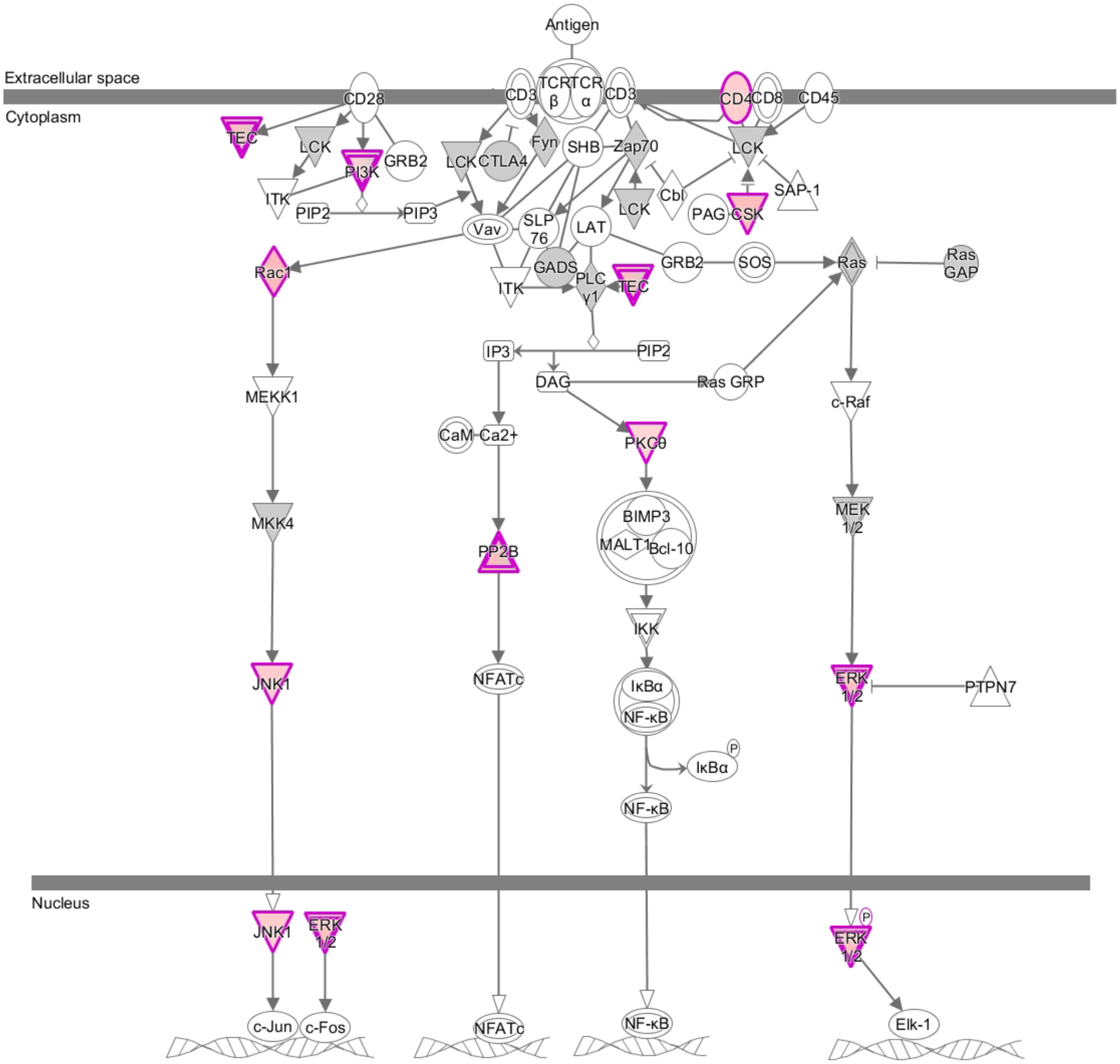


**Supplemental Figure VIII: T cell receptor signaling canonical pathway from Ingenuity Pathway Analysis.** Nodes represent gene symbol name, corresponding to protein measured. The intensity of the node color, ranging from light purple to dark red, indicates degree of significance of FDR p-value, with dark red indicating a higher degree of significance. White nodes represent genes in the IPA canonical pathway that were not significant or not measured. The node shapes denote complex/group (◎), chemical (⬭), cytokine (□), disease (✚), enzyme (◊), G-protein coupled receptor (▯), growth factor (⬚), ion channel (▯), kinase (▽), ligand-dependent nuclear receptor (▭), peptidase (◇),

phosphatase (△), transcription regulator (⬭), transmembrane receptor (⬯), transporter (⏢), and other (○).

**Sphingosine-1-phosphate Signaling**

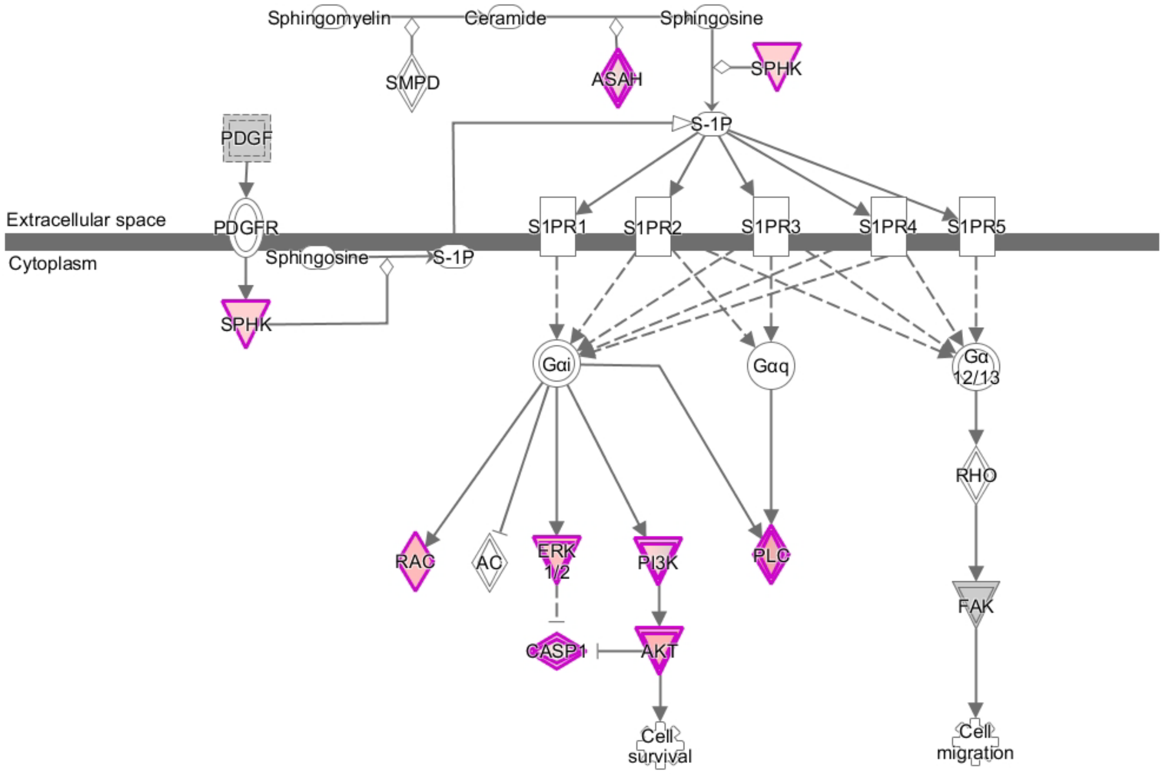


**Supplemental Figure IX: Sphingosine-1-phosphate signaling canonical pathway from Ingenuity Pathway Analysis.** Nodes represent gene symbol name, corresponding to protein measured. The intensity of the node color, ranging from light purple to dark red, indicates degree of significance of FDR p-value, with dark red indicating a higher degree of significance. White nodes represent genes in the IPA canonical pathway that were not significant or not measured. The node shapes denote complex/group ( ), chemical ( ), cytokine ( ), disease ( ), enzyme ( ), G-protein coupled receptor ( ), growth factor ( ), ion channel ( ), kinase ( ), ligand-dependent nuclear receptor ( ), peptidase ( ), phosphatase ( ), transcription regulator ( ), transmembrane receptor ( ), transporter ( ), and other ( ).

## PI3K/AKT Signaling

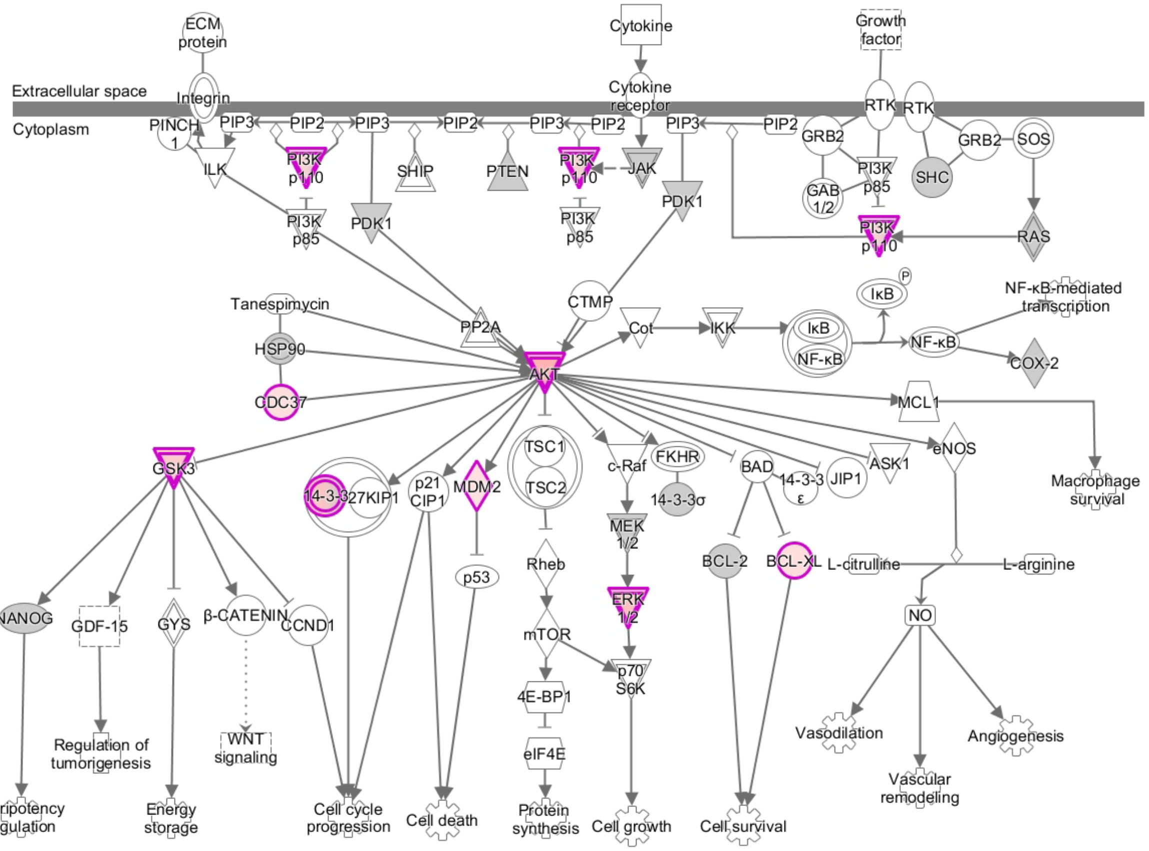


**Supplemental Figure X: PI3K/AKT signaling canonical pathway from Ingenuity Pathway Analysis.** Nodes represent gene symbol name, corresponding to protein measured. The intensity of the node color, ranging from light purple to dark red, indicates degree of significance of FDR p-value, with dark red indicating a higher degree of significance. White nodes represent genes in the IPA canonical pathway that were not significant or not measured. The node shapes denote complex/group ( ), chemical ( ), cytokine ( ), disease ( ), enzyme ( ), G-protein coupled receptor ( ), growth factor ( ), ion channel ( ), kinase ( ), ligand-dependent nuclear receptor ( ), peptidase ( ), phosphatase ( ), transcription regulator ( ), transmembrane receptor ( ), transporter ( ), and other ( ).

**TREM1 Signaling**

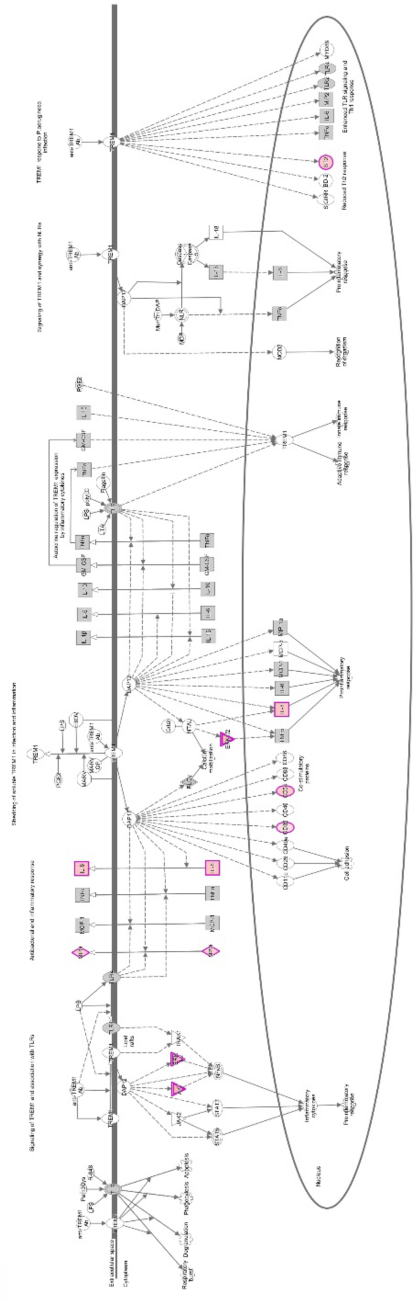

**Supplemental Figure XI: TREM1 signaling canonical pathway from Ingenuity Pathway Analysis.** Nodes represent gene symbol name, corresponding to protein measured. The intensity of the node color, ranging from light purple to dark red, indicates degree of significance of FDR p-value, with dark red indicating a higher degree of significance. White nodes represent genes in the IPA canonical pathway that were not significant or not measured. The node shapes denote complex/group (◎), chemical (⬭

), cytokine ( ), disease ( ), enzyme ( ), G-protein coupled receptor ( ), growth factor ( ), ion channel ( ), kinase ( ), ligand-dependent nuclear receptor ( ), peptidase ( ), phosphatase ( ), transcription regulator ( ), transmembrane receptor ( ), transporter ( ), and other ( ).

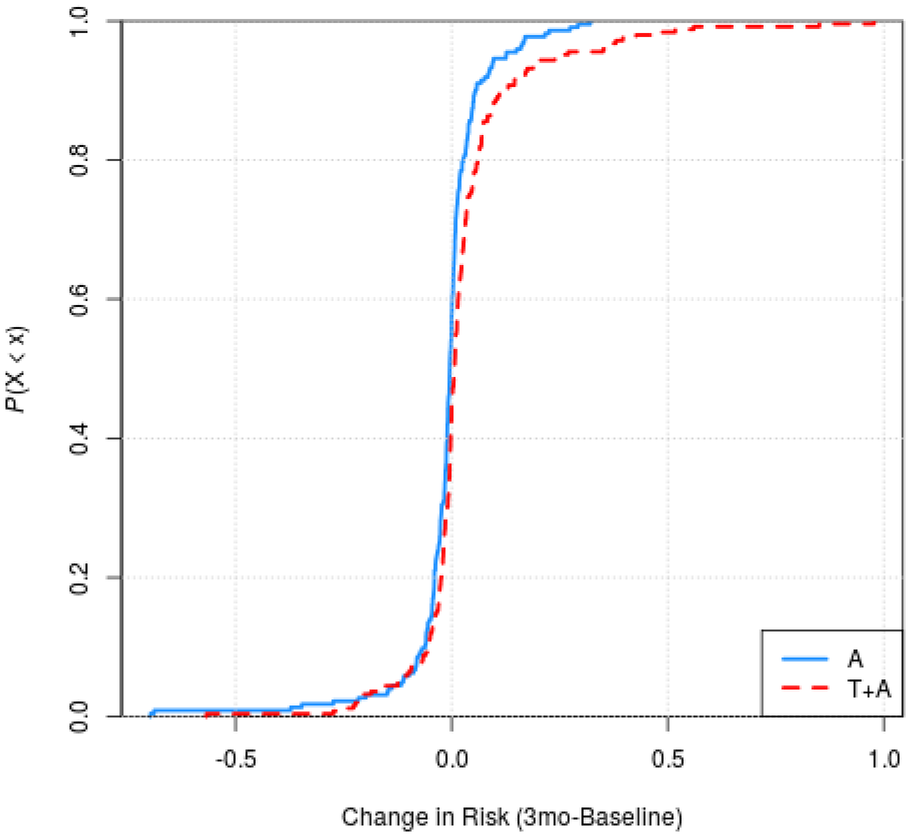


**Supplemental Figure XII: Cumulative distribution function for within-participants change in risk for the T+A group (red) and the A group (blue) showing a small but relatively consistent upward shift in risk in the T+A group for the majority of the study population.** T = torcetrapib; A = atorvastatin.

**Supplemental References**

**1.** Ganz P, Heidecker B, Hveem K, et al. Development and Validation of a Protein-Based Risk Score for Cardiovascular Outcomes Among Patients With Stable Coronary Heart Disease. *JAMA.* 2016;315:2532-2541.
